# Alternative pathways utilize or circumvent putrescine for biosynthesis of putrescine-containing rhizoferrin

**DOI:** 10.1074/jbc.RA120.016738

**Published:** 2020-12-10

**Authors:** Bin Li, Xiaoyi Deng, Sok Ho Kim, Leann Buhrow, Diana R. Tomchick, Margaret A. Phillips, Anthony J. Michael

**Affiliations:** 1 Department of Biochemistry, UT Southwestern Medical Center, Dallas, Texas, USA; 2 Department of Biophysics, UT Southwestern Medical Center, Dallas, Texas, USA

**Keywords:** rhizoferrin, putrescine, ornithine, *N*-citrylornithine, *Francisella*, *Ralstonia*, *Legionella*, siderophore, polyamine, decarboxylase, ADC, L-arginine decarboxylase, AR-fold, alanine racemase-fold, CANSDC, carboxynorspermidine decarboxylase, CASDC, carboxyspermidine decarboxylase, DAPDC, *meso*-diaminopimelate decarboxylase, D-ODC, D-ornithine decarboxylase, L-ODC, L-ornithine decarboxylase, L/ODC, bifunctional lysine/ornithine decarboxylase, MFS, major facilitator superfamily, NIS, nonribosomal peptide synthetase-independent siderophore

## Abstract

The siderophore rhizoferrin (*N*^1^,*N*^4^-dicitrylputrescine) is produced in fungi and bacteria to scavenge iron. Putrescine-producing bacterium *Ralstonia pickettii* synthesizes rhizoferrin and encodes a single nonribosomal peptide synthetase-independent siderophore (NIS) synthetase. From biosynthetic logic, we hypothesized that this single enzyme is sufficient for rhizoferrin biosynthesis. We confirmed this by expression of *R. pickettii* NIS synthetase in *Escherichia coli*, resulting in rhizoferrin production. This was further confirmed *in vitro* using the recombinant NIS synthetase, synthesizing rhizoferrin from putrescine and citrate. Heterologous expression of homologous *lbtA* from *Legionella pneumophila*, required for rhizoferrin biosynthesis in that species, produced siderophore activity in *E. coli*. Rhizoferrin is also synthesized by *Francisella tularensis* and *Francisella novicida*, but unlike *R. pickettii* or *L. pneumophila*, *Francisella* species lack putrescine biosynthetic pathways because of genomic decay. *Francisella* encodes a NIS synthetase FslA/FigA and an ornithine decarboxylase homolog FslC/FigC, required for rhizoferrin biosynthesis. Ornithine decarboxylase produces putrescine from ornithine, but we show here *in vitro* that FigA synthesizes *N*-citrylornithine, and FigC is an *N*-citrylornithine decarboxylase that together synthesize rhizoferrin without using putrescine. We co-expressed *F. novicida figA* and *figC* in *E. coli* and produced rhizoferrin. A 2.1 Å X-ray crystal structure of the FigC *N*-citrylornithine decarboxylase reveals how the larger substrate is accommodated and how active site residues have changed to recognize *N*-citrylornithine. FigC belongs to a new subfamily of alanine racemase-fold PLP-dependent decarboxylases that are not involved in polyamine biosynthesis. These data reveal a natural product biosynthetic workaround that evolved to bypass a missing precursor and re-establish it in the final structure.

Almost all organisms require iron for growth (1), and many microorganisms obtain iron from the environment by synthesizing, secreting, and retrieving small molecular weight iron chelators known as siderophores (2). Siderophores employ hydroxamate, catecholate, or α-hydroxycarboxylate functional groups to bind Fe^3+^ (3). Biosynthesis of siderophores employs either the intensively studied nonribosomal peptide synthetase siderophore synthetases (4) or the more recently characterized nonribosomal peptide synthetase–independent siderophore (NIS) synthetases (5, 6). Diverse siderophores utilize primary metabolites of the polyamine family 1,3-diaminopropane, putrescine (1,4-diaminobutane), cadaverine (1,5-diaminpentane), norspermidine (*N*-aminopropyl-1,3-diaminopropane), spermidine (*N*-aminopropylputrescine), and homospermidine (*N*-aminobutylputrescine) (7, 8), as structural backbones onto which the iron-binding hydroxamate, catecholate, or α-hydroxycarboxylate functional groups are appended via amide linkages. Examples of spermidine-containing siderophores are the dicatecholate siderophore petrobactin from the anthrax agent *Bacillus anthracis* (9) and the tricatecholate siderophore agrobactin from the plant pathogen *Agrobacterium tumefaciens* (10). Homospermidine is found in the petrobactin structural analog rhodopetrobactin from the environmental bacterium *Rhodopseudomonas palustris* (11). Norspermidine is found in the tricatecholate siderophore vibriobactin produced by the cholera agent *Vibrio cholerae* (12).

The diamine putrescine, biosynthetic precursor of spermidine and homospermidine, is found in a range of cyclic and linear hydroxamate-based siderophores such as desferrioxamine, putrebactin, and avaroferrin (13, 14). It is also found as a monocatecholate siderophore, *e.g.*, aminochelin (15), and the dicatecholate siderophore photobactin (16). Putrescine is also found in the simple polycarboxylate siderophore rhizoferrin (*N*^1^,*N*^4^-dicitrylputrescine), consisting of two citrate molecules linked to a putrescine backbone (17). Rhizoferrin was first characterized from the zygomycete fungus *Rhizopus microspores*, and the fungal rhizoferrin is produced as the R,R-rhizoferrin enantiomer (18, 19). Fungal rhizoferrin was found to be synthesized by a single NIS synthetase in *Rhizopus delemar* (20). Subsequent to the discovery of fungally produced rhizoferrin, it was then found in the β-proteobacterium *Ralstonia pickettii*, as the S,S-rhizoferrin enantiomer (21). The polyamines produced by *R. pickettii* are putrescine and 2-hydroxyputrescine (22, 23), but nothing is known about how rhizoferrin is produced in this species. Recently, the citrate-containing siderophore of the Legionnaires’ disease agent *Legionella pneumophila* was shown to be rhizoferrin (24), and it was previously demonstrated that production of the siderophore is dependent on the NIS synthetase LbtA and major facilitator superfamily (MFS)–type transporter LbtB (25). Putrescine and homospermidine are produced by *L. pneumophila* (26).

Intriguingly, rhizoferrin is also produced by the LVS and SCHU S4 strains of the tularemia agent *Francisella tularensis* (27, 28, 29). The first sequenced genome of *F. tularensis*, the virulent SCHU S4 strain, revealed that the pathogen has a small genome of 1.89 Mbp that is undergoing decay associated with an intracellular lifestyle (30). More than 10% of the coding sequences contain insertion-deletion or substitution mutations resulting in loss of metabolic pathways. Closely related species *Francisella novicida* does not infect humans except opportunistically and is 98% identical to *F. tularensis* (31, 32). The ancestor of *Francisella* probably synthesized putrescine via the activities of arginine decarboxylase, agmatine deiminase, and *N*-carbamoylputrescine amidohydrolase; however, these genes have become disrupted in the case of *F. tularensis* or lost in *F. novicida* (30, 33). The *fslA*/*figA*-encoded NIS synthetase is required for rhizoferrin production in *F. tularensis* and *F. novicida* (27, 28). In addition, MFS-type transporter gene *figB* and *figC*-encoded alanine racemase (AR)-fold PLP-dependent decarboxylase, homologous to ornithine decarboxylase (OD), are required for rhizoferrin production in *F. novicida* (34). As *F. novicida* has lost all putrescine biosynthetic genes, we sought to determine how rhizoferrin is produced by this species. During the course of our study, another recent study demonstrated that a *figC* deletion mutant of *F. tularensis* accumulates *N*-citrylornithine, indicating that *figC* encodes an *N*-citrylornithine decarboxylase (35). We also sought to determine how rhizoferrin is produced by *R. pickettii* and *L. pneumophila*, which synthesize putrescine. To elucidate the *R. pickettii*, *L. pneumophila*, and *F. novicida* biosynthetic pathways, we expressed relevant genes in *Escherichia coli*, and we assayed purified recombinant proteins *in vitro*. The X-ray crystal structure of the PLP-dependent decarboxylase FigC, which we confirmed *in vitro* to be an *N*-citrylornithine decarboxylase, was determined.

## Results

### Putrescine-dependent bacterial rhizoferrin biosynthesis

**Figure 1:**
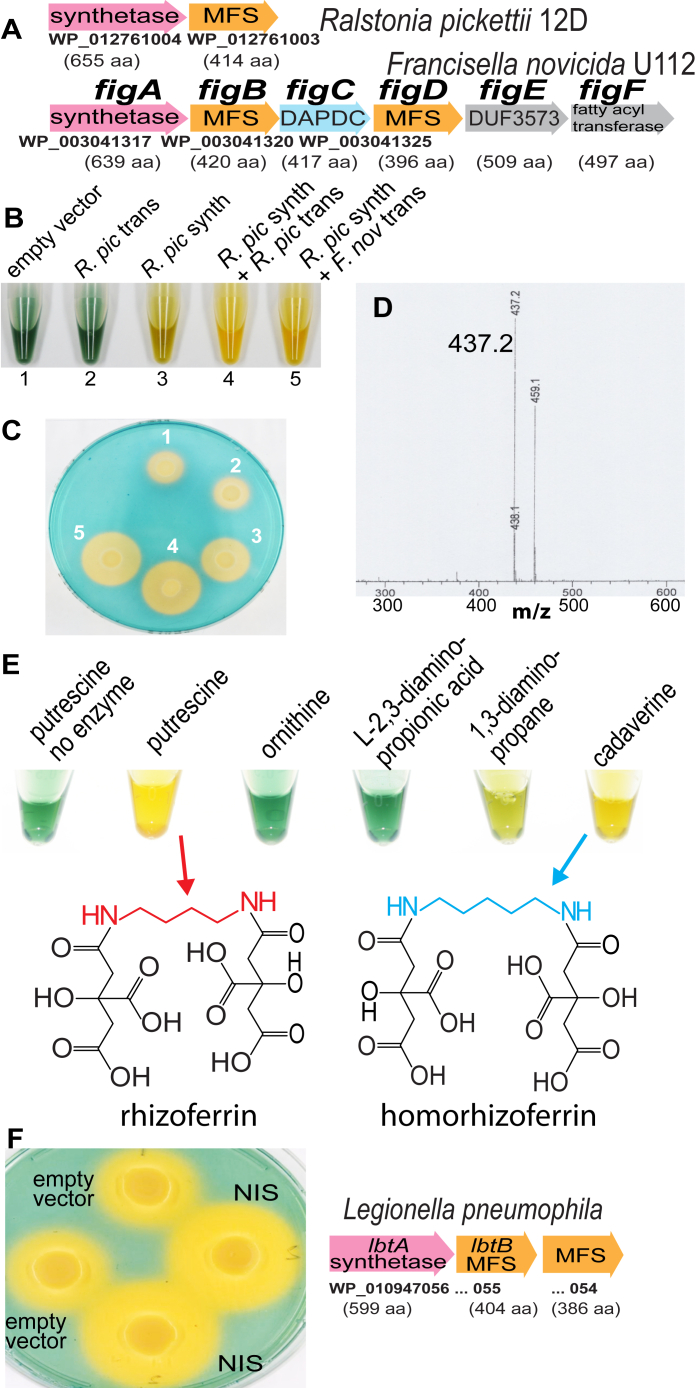
**Production of rhizoferrin by the *Ralstonia pickettii* NIS synthetase in *E. coli* and *in vitro*.***A*, gene clusters containing the NIS synthetase and major facilitator superfamily (MFS) transporter in *Ralstonia pickettii* and homologs in *Francisella novicida*. GenBank protein accession numbers are shown below each ORF, and length of each putative encoded protein product is shown in parentheses. DAPDC, a PLP-dependent decarboxylase annotated by GenBank as *meso*-diaminopimelate decarboxylase. *B*, CAS activity in liquid culture supernatant of *E. coli* BL21 containing pETDuet-1 expressing either the *R. pickettii* NIS synthetase (*R. pic* synth, WP_012761004), MFS transporter (*R. pic* trans, WP_012761003), or both (*R.pic* synth + *R. pic* trans), or the *R. pickettii* NIS synthetase and *F. novicida figB* MFS transporter WP_003041320 (*R. pic* synth + *F. nov* trans). *C*, CAS activity of the strains shown in Figure 1*B* after growth on CAS plates. *D*, mass spectrum of partially purified siderophore from LB growth medium culture supernatant of *E. coli* BL21 expressing the *R. pickettii* NIS synthetase and MFS transporter from pETDuet-1. A mass corresponding to rhizoferrin (m/z 437) and its sodium adduct (m/z 459) are detected. *E*, *in vitro* siderophore biosynthesis and substrate preference of the purified recombinant *R. pickettii* NIS synthetase. CAS siderophore detection from *in vitro* assays of 1.5 μM NIS synthetase with 10 mM indicated substrates. The relevant masses of rhizoferrin and homorhizoferrin detected in these assays are shown in Figure S1. *F*, siderophore detection on CAS plates after growth of *E. coli* containing pETDuet-1 co-expressing the *Legionella pneumophila figA* homolog NIS synthetase (NIS, WP_010947056, first 19 amino acids removed) with *R. pickettii* MFS transporter (WP_012761003) or empty vector (replicates). The corresponding *L. pneumophila* NIS synthetase gene cluster is shown.

The environmental β-proteobacterium *R. pickettii* produces rhizoferrin and encodes (Fig. 1*A*) a single NIS synthetase of 655 amino acids. Immediately downstream is a 414 amino acid ORF encoding a putative MFS transporter protein. To determine whether the *R. pickettii* NIS synthetase and MFS transporter are responsible for rhizoferrin production and sufficient to synthesize and export it when expressed heterologously in *E. coli* BL21, the genes were expressed individually or together from the expression vector pETDuet-1. Siderophore production resulting from growth of liquid cultures (Fig. 1*B*) or from solid agar plates (Fig. 1*C*) was detected using the Chrome Azurol S (CAS) reagent, siderophore production being visualized by formation of an intense yellow coloration. Expression of the *R. pickettii* NIS synthetase alone in *E. coli* was sufficient to produce a positive reaction with the CAS reagent, which was more intense when the *R. pickettii* MFS transporter or the *F. novicida* FigB MFS transporter was co-expressed. After partial purification of the supernatant from liquid cultures of *E. coli* BL21 co-expressing the *R. pickettii* NIS synthetase and MFS transporter and analysis by LC-MS, a mass corresponding to rhizoferrin (m/z 437.2) was detected that was absent in the control culture containing the empty plasmid (Fig. 1*D*). The presence of CAS-positive siderophore in the external medium, when *E. coli* BL21 expressing only the NIS synthetase was grown, indicates that rhizoferrin is exported by an *E. coli* efflux transporter. A more intense CAS reaction when the *R. pickettii* or *F. novicida* FigB MFS was co-expressed with the NIS synthetase may be because of greater affinity of the *R. picketti* or *F. novicida* transporters for rhizoferrin, or it may be because of their potentially higher expression levels compared with the native *E. coli* transporter(s). An alignment of the only close *E. coli* homolog, the MFS transporter MdtG (NP_415571), with the *R. pickettii* MFS, *F. novicida* FigB, and *L. pneumophila* LbtB transporters is shown in Figure S1A. A maximum likelihood phylogenetic tree indicates that *F. novicida* FigB and *L. pneumophila* LbtB transporters are more closely related compared with the *R. pickettii* MFS or *E. coli* MdtG proteins (Fig. S1B). The *E. coli* MdtG efflux transporter is involved in fluoroquinolone resistance (36), and it is notable (Fig. S1C) that there are two internal amine groups separated by three carbons in the structure of quinolone antibiotics such as ciprofloxacin (37).

To assess the substrate specificity of the *R. pickettii* NIS synthetase, the recombinant his-tagged purified protein was assayed *in vitro* with citrate and different diamine and amino acid cosubstrates and siderophore production then detected with the CAS reagent (Fig. 1*E*). Putrescine and cadaverine produced a strong CAS reaction, a less intense reaction was detected with 1,3-diaminopropane, but no reaction was detected with L-ornithine or L-2,3-diaminopropionic acid. Analysis by LC-MS (Fig. S2) of the *in vitro* reaction products produced with putrescine or cadaverine revealed masses for rhizoferrin (m/z 437.2) or homorhizoferrin (m/z 451.2), respectively (Fig. 1*E*).

As a dedicated efflux transporter did not appear to be required for excretion of heterologously produced rhizoferrin from *E. coli* BL21, we expressed in *E. coli* the *L. pneumophila* gene encoding the LbtA NIS synthetase that is required for rhizoferrin biosynthesis in that species. Expression of the *L. pneumophila* subsp. pneumophila str. Philadelphia 1 *lbtA* NIS synthetase (599 amino acids) alone from pETDuet-1 in *E. coli* did not result in siderophore production, as detected by the CAS reagent after growth on solid agar plates. We then noted that the *lbtA* ORF from the 130b strain of *L. pneumophila* used in the original study (25) lacked 19 amino acids from the N-terminus relative to the Philadelphia 1 strain. After removal of the first 19 amino acids from the Philadelphia 1 LbtA NIS synthetase, which consisted of the region up to the first internal ATG codon, expression of the shortened version in *E. coli* BL21 then resulted in siderophore production (Fig. 1*F*). It is noteworthy that *R. pickettii* likely synthesizes putrescine from ornithine using an aspartate aminotransferase-fold ODC (ACS63448, 759 a.a.) that is a close homolog (95% amino acid identity) of the characterized (38) *Ralstonia solanacearum* ODC (CAD16072, 759 a.a.). In contrast, *L. pneumophila* likely synthesizes putrescine from arginine using an AR-fold arginine decarboxylase, agmatine deiminase, and *N*-carbamoylputrescine amidohydrolase (see correction to [39]).

### Putrescine circumventing, ornithine-dependent bacterial rhizoferrin biosynthesis

**Figure 2:**
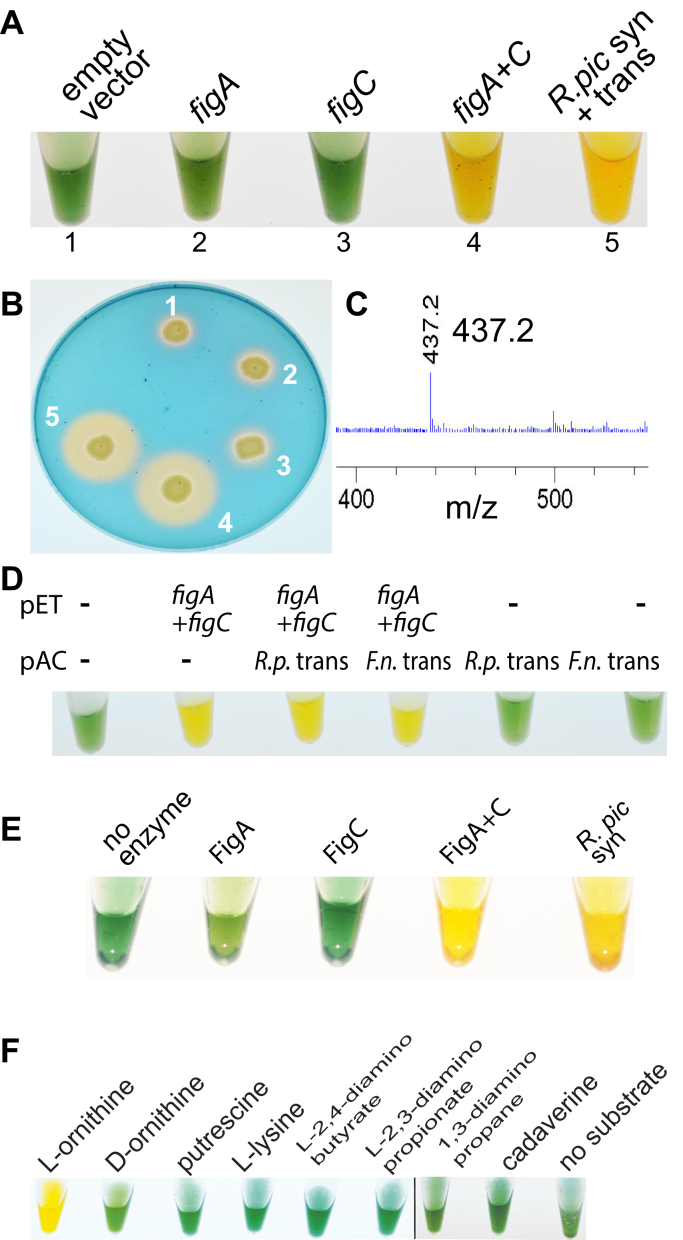
***F. novicidia* FigC is required for rhizoferrin production by FigA**. *A*, CAS reactive siderophore production in culture supernatant by *E. coli* BL21 expressing either the *F. novicida* NIS synthetase *figA* (WP_003041317), PLP-dependent decarboxylase *figC* (WP_003041325) or *figA* + *figC*, from pETDuet-1. A positive control of *E. coli* BL21 coexpressing the *R. pickettii figA* homolog NIS synthetase and MFS transporter from pETDuet-1 is shown (*R. pic* syn + trans). *B*, the same strains shown in (*A*) grown on CAS plates. *C*, mass spectrum of partially purified siderophore from the culture supernatant of *E. coli* BL21 coexpressing the *F. novicida figA* + *figC* from pETDuet-1. The prominent peak at 437.2 corresponds to rhizoferrin. *D*, the effect of coexpressing the *F. novicida figB* MFS transporter (F.n. trans) or *R. pickettii* MFS transporter (WP_012761003, R.p. trans) from pACYCDuet-1 (pAC), on siderophore production from expression of *F. novicida figA* + *figC* (pETDuet-1, pET) in *E. coli* BL21. Presence of siderophore in the culture supernatant was detected by CAS reactivity. *E*, CAS siderophore detection from *in vitro* assays with purified recombinant *F. novicida* FigA, FigC, FigA + FigC or *R. pickettii* NIS synthetase (*R. pic* syn). Enzymes are present at 2 μM with 3 mM Na-citrate and 10 mM L-ornithine (10 mM putrescine for the *R. pickettii* NIS synthetase). *F*, CAS siderophore detection from *in vitro* assays with purified recombinant *F. novicida* FigA + FigC (2 μM) with 3.0 mM Na-citrate and 10 mM indicated amines or amino acids. CAS, Chrome Azurol S; MFS, major facilitator superfamily.

In contrast to rhizoferrin biosynthesis in *R. pickettii* and *L. pneumophila*, *F. novicida* requires not only the encoded NIS synthetase FigA and MFS transporter FigB but also a homolog (FigC) of the AR-fold PLP-dependent decarboxylase family that includes L-ornithine decarboxylase (L-ODC), an enzyme that produces putrescine from L-ornithine (34). We expressed separately either the *F. novicida figA* NIS synthetase gene or the *figC* decarboxylase, from pETDuet-1 in *E. coli* BL21 grown in liquid culture or on solid agar plates, but no siderophore production was detected using the CAS reagent (Fig. 2, *A*–*B*). However, co-expression of *figA* and *figC* resulted in a strong positive CAS reaction comparable to the reaction produced by co-expression of the *R. pickettii* NIS synthetase and MFS transporter. Partial purification of the supernatant from the *E. coli* liquid culture co-expressing the *F. novicida figA* and *figC* genes and analysis by LC-MS (Fig. 2*C*) revealed a mass corresponding to rhizoferrin (m/z 437.2). To determine whether co-expression of the *F. novicida figB* or *R. pickettii* MFS transporters together with *figA* and *figC* would increase siderophore production from *E. coli*, we co-expressed *figA* and *figC* from pETDuet-1 and *figB* or the *R. pickettii* MFS transporter from pACYCDuet-1 (Fig. 2*D*). Co-expression of either transporter noticeably increased the intensity of the CAS reagent positive reaction.

We reasoned that if FigC was an L-ODC producing putrescine from ornithine, then *figC* would not be required for heterologous rhizoferrin biosynthesis in *E. coli*, as there is already abundant putrescine present. Therefore, the most likely explanation for the requirement for *figC* would be that FigA conjugates L-ornithine to citrate to form *N*-citrylornithine, which is then decarboxylated by FigC to form *N*-citrylputrescine. Recombinant FigA and FigC proteins were purified and assayed *in vitro* with the CAS reagent, and assay reactions contained 2 mM Na-citrate and 2 mM L-ornithine (Fig. 2*E*). A slight positive reaction was observed with FigA alone, but an intense reaction was observed with FigA and FigC together, comparable with the reaction produced by the *R. pickettii-*purified recombinant NIS synthetase (Fig. 2*E*). Although FigA and FigC together produced a positive reaction with L-ornithine, no reaction was seen with D-ornithine, L-lysine, putrescine, cadaverine, 1,3-diaminopropane, L-2,4-diaminobutyrate, or L-2,3-diaminopropionate (Fig. 2*F*). When the reaction products from FigA alone assayed with citrate and L-ornithine assay were analyzed by LC-MS, a mass for *N*-citrylornithine was detected (m/z 307.1). With FigA and FigC together, a mass for rhizoferrin (m/z 437.1) was detected (Fig. S3).

**Figure 3:**
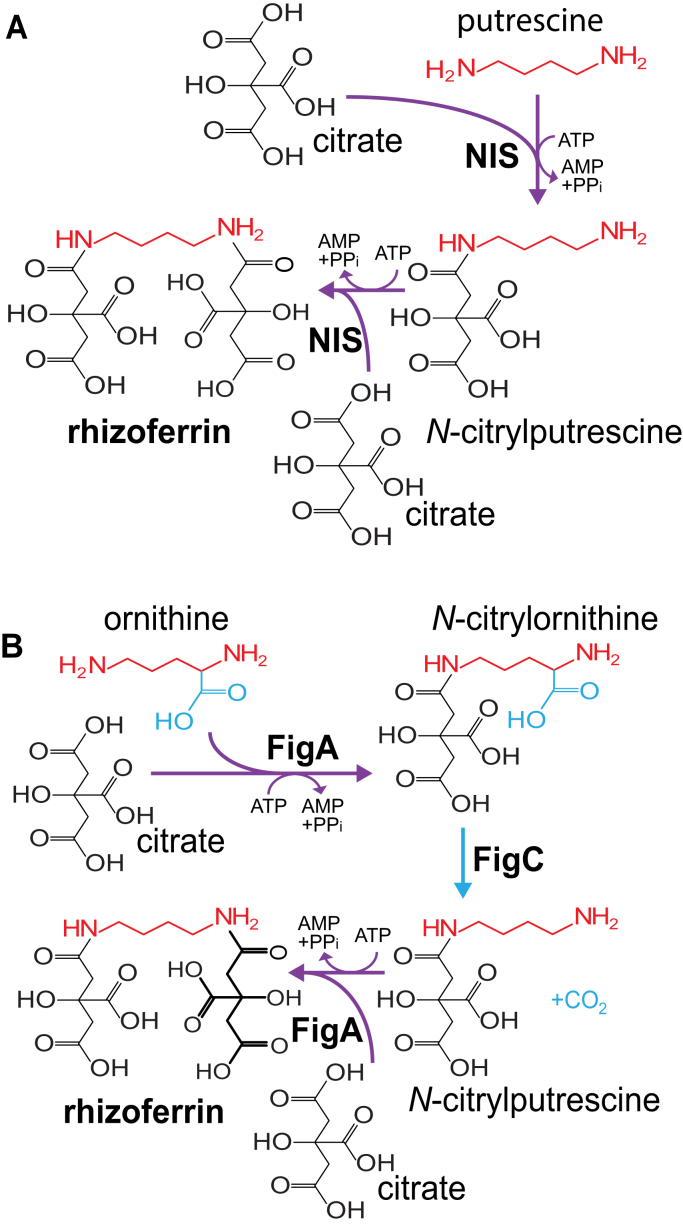
**Proposed pathways for rhizoferrin biosynthesis in (*A*) *R. pickettii* (and *L. pneumophila*) and (*B*) *F. novicida* (and *F. tularensis*).** NIS, *R. pickettii* NIS synthetase. NIS, nonribosomal peptide synthetase-independent siderophore.

The requirement for *F. novicida figC* in heterologous rhizoferrin biosynthesis in *E. coli*, the fact that FigA can use only L-ornithine and not putrescine *in vitro* for rhizoferrin biosynthesis, and the need for FigC in the conversion of *N*-citrylornithine to rhizoferrin *in vitro* confirms that FigC is an *N*-citrylornithine decarboxylase and not an L-ornithine decarboxylase. On the basis of these findings, we propose the two alternative bacterial rhizoferrin biosynthetic pathways depicted in Figure 3. The *R. pickettii* NIS synthetase, and by extension the *L. pneumonphila* LbtA NIS synthetase, is alone sufficient for rhizoferrin biosynthesis via *N*-citrylputrescine (Fig. 3*A*). In contrast, the *F. novicida* FigA NIS synthetase produces *N*-citrylornithine, which is then decarboxylated to *N*-citrylputrescine by FigC, and another citrate condensed to *N*-citrylputrescine by FigA to produce rhizoferrin (Fig. 3*B*). Recently, Ramakrishnan *et al.* (35) have shown that a *figC* deletion mutant of *F. tularensis* LVS accumulates *N*-citrylornithine.

### X-ray crystal structure determination of the *N*-citrylornithine decarboxylase FigC

**Figure 4:**
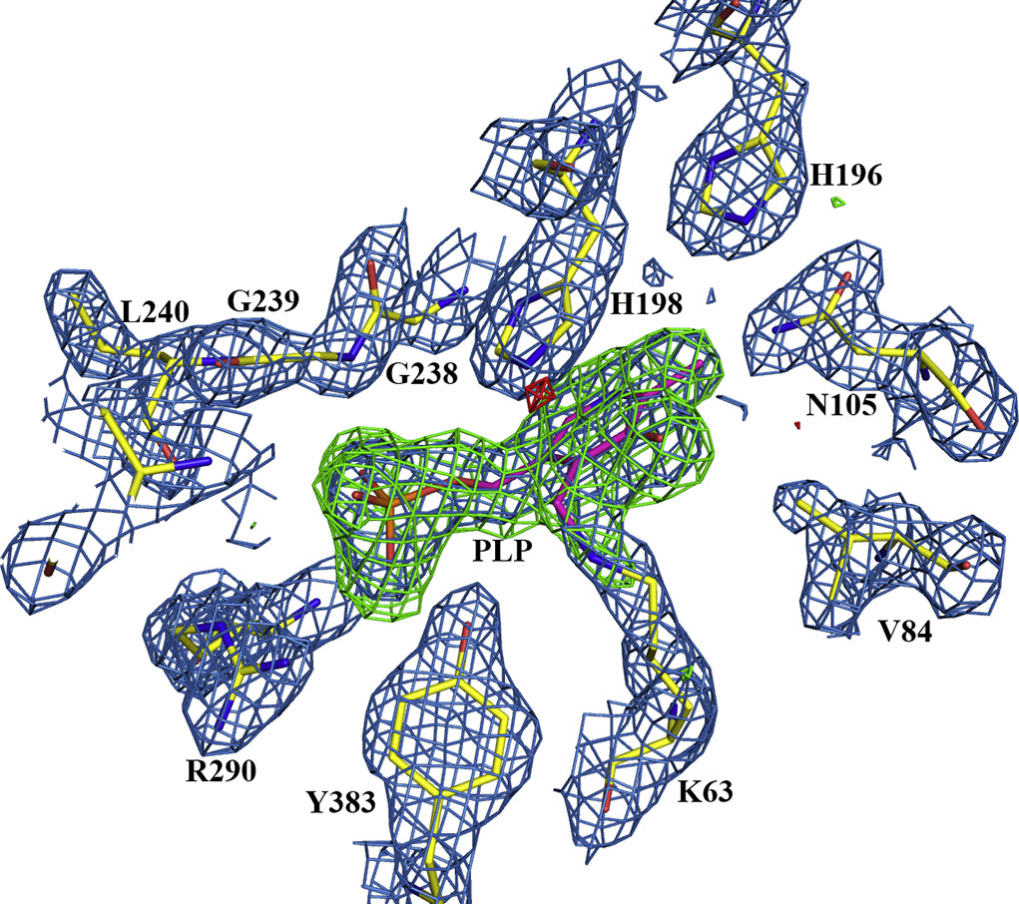
**Diagram of the FigC active site showing the electron density from the structure refinement.** The 2fofc map of the final refined model contoured at 1.5 sigma is shown in *blue*. The fofc map generated before PLP was modeled into the structure is also shown; *green* shows positive density and red is negative density, contoured at +3 and −3 sigma respectively. *Yellow* is the refined protein model, while PLP is in *pink*.

**Figure 5:**
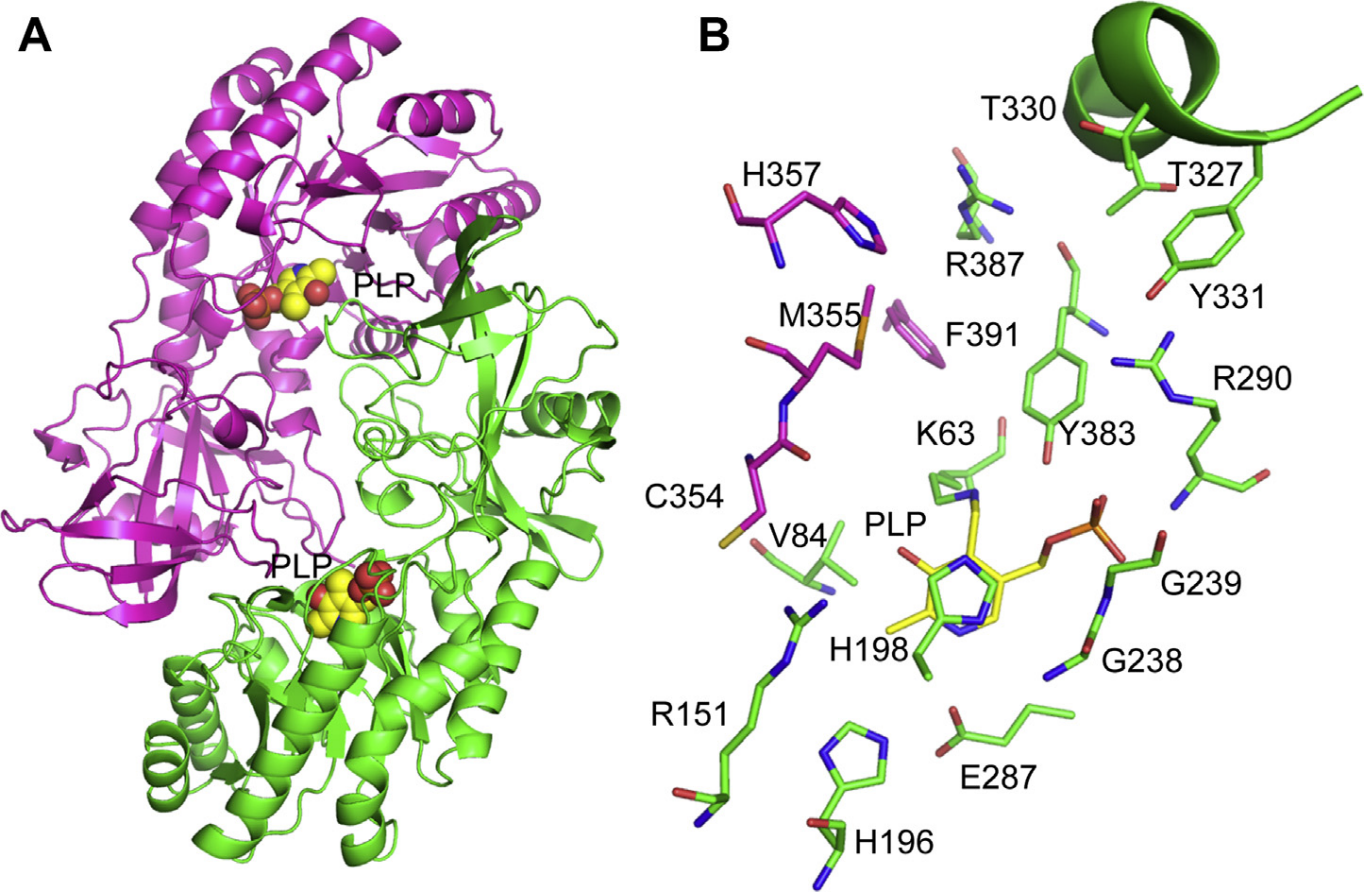
**Structure of *N*-citrylornithine decarboxylase FigC solved to 2.1 Å resolution.***A*, cartoon diagram showing the dimeric structure with subunit (a) colored in *pink* and subunit (b) colored in *green*. Bound PLP is shown as balls colored with carbons in *yellow*, oxygen in *red*, and nitrogen in *blue*. *B*, FigC shared active site structure with subunits and PLP colored as in (*A*). The specificity helix is drawn as a cartoon.

**Figure 6:**
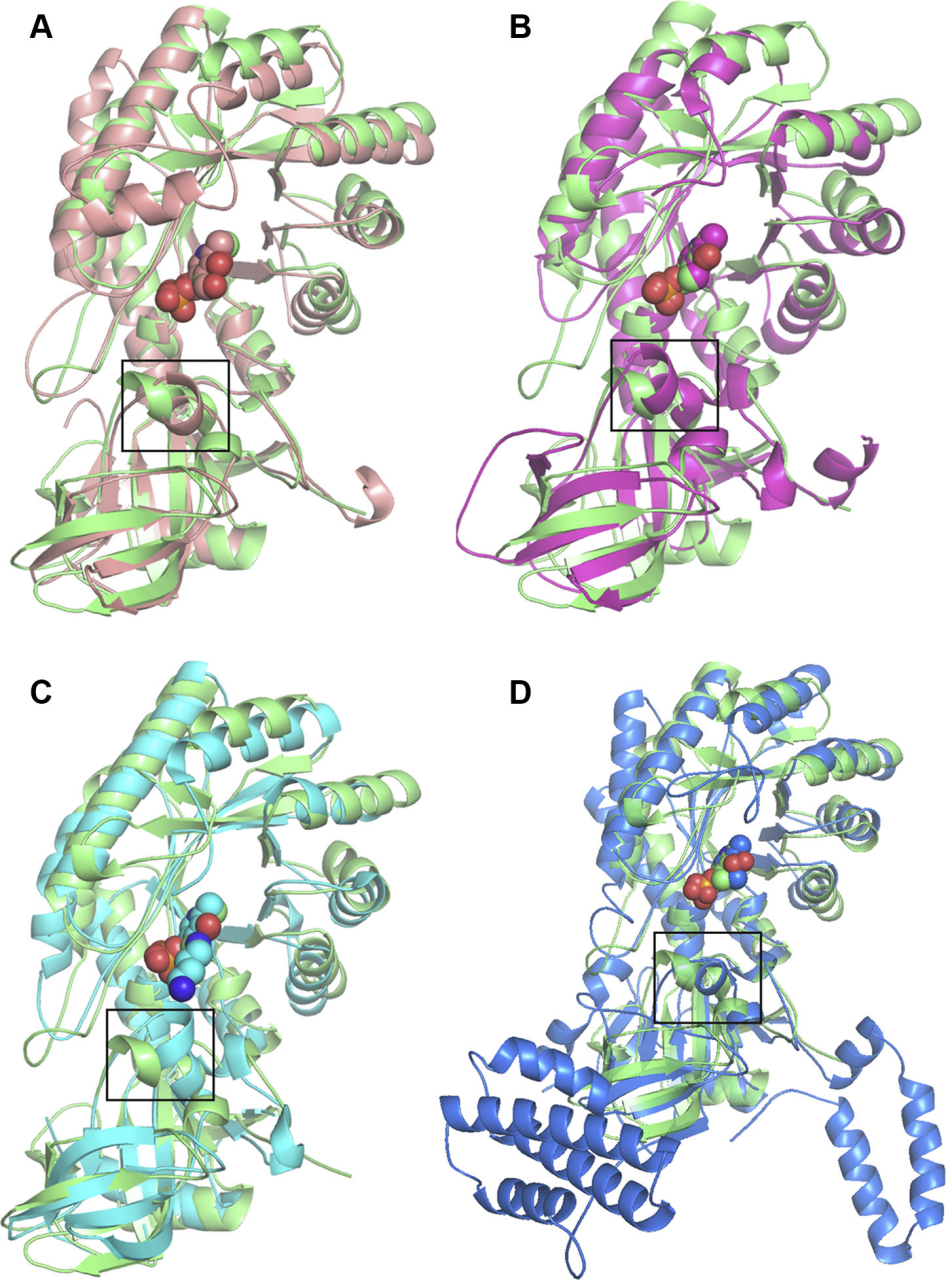
**Structural alignment of FigC with other PLP-dependent enzymes from the β/α structural fold.***A*, *Methanocaldococcus jannaschii meso*-diaminopimelate decarboxylase (1TWI) displayed in *salmon*. The RMS was 2.1 Å over 3866 atoms. *B*, *Campylobacter jejuni* carboxynorspermidine decarboxylase (3N29) displayed in *magenta*. The RMS was 2.2 Å over 3265 atoms. *C*, *Vibrio vulnificus* lysine/ornithine decarboxylase in complex with putrescine (2PLJ) displayed in *turquoise* for carbons and *dark blue* for the terminal amines. The RMS was 2.1 Å over 3147 atoms. *D*, *V. vulnificus* arginine decarboxylase (3N2O) displayed in *dark blue*. The RMS was 3.3 Å over 3050 atoms. Secondary structures are depicted as a cartoon and loops have been smoothed to simplify the display. FigC is shown in *green* in all structures, and the orientation is identical in all structures. The black box in each figure shows the specificity helix. PLP is shown as balls and is displayed for all structures, in addition the putrescine ligand is displayed for 2PLJ in panel C.

Given the novel substrate preference of the FigC decarboxylase for *N*-citrylornithine, we solved the X-ray crystal structure of FigC and compared it with other AR-fold PLP-dependent decarboxylases. The X-ray crystal structure was solved to 2.1 Å resolution by single-wavelength anomalous diffraction using incorporated selenomethionine to phase the structure (Table S1). Two functional dimers were found in the asymmetric unit (four monomers), and all four active sites showed good density for the cofactor PLP (Fig. 4). FigC is composed of an N-terminal β/α-barrel domain and a C-terminal β-barrel domain (Fig. 5*A*), typical of the AR-fold PLP-dependent decarboxylases. The FigC structure exhibits strong similarity to other structures within the family representing diverse substrate specificities, including *meso*-diaminopimelate decarboxylase (DAPDC) from the euryarchaeote *Methanocaldococcus jannaschii*, carboxyspermidine decarboxylase (CASDC) from *Campylobacter jejuni*, lysine/ornithine decarboxylase (L/ODC) from *Vibrio vulnificus*, and arginine decarboxylase (ADC) from *V. vulnificus* (Fig. 6). An internal insertion and C-terminal extension in ADC confers a tetrameric rather than dimeric structure (47).

The FigC active site is positioned at both the monomer domain interface and the dimer interface between the N-terminal β/α-barrel of one subunit and the β-barrel domain of the second (Figs. 4 and 5*B*). A Schiff base is formed between the PLP cofactor and the conserved catalytic lysine (K63 in FigC). This residue has been shown previously to be essential for facilitation of the transamination step that accompanies substrate binding and product release and also for decarboxylation in eukaryotic ODCs (40, 41). Additional conserved residues with demonstrated roles in the catalytic cycle in these eukaryotic ODC enzymes are also observed to make key contacts with PLP in the FigC structure. These include E287, which interacts with the pyridine nitrogen of PLP to facilitate decarboxylation by stabilizing the carbanion intermediate (42); the glycine loop (G237–239) and R290, which form important backbone amide interactions with the phosphate of PLP contributing to tight binding (43); H198, which stacks against the PLP ring, and R151 positioned near the PLP hydroxyl. Additionally, C354, shown to function as a catalytic base in eukaryotic ODCs (41, 44), is also conserved in FigC and contributes to the active site from across the dimer interface.

### Molecular docking of PLP-*N*-citrylputrescine into the FigC active site

**Figure 7:**
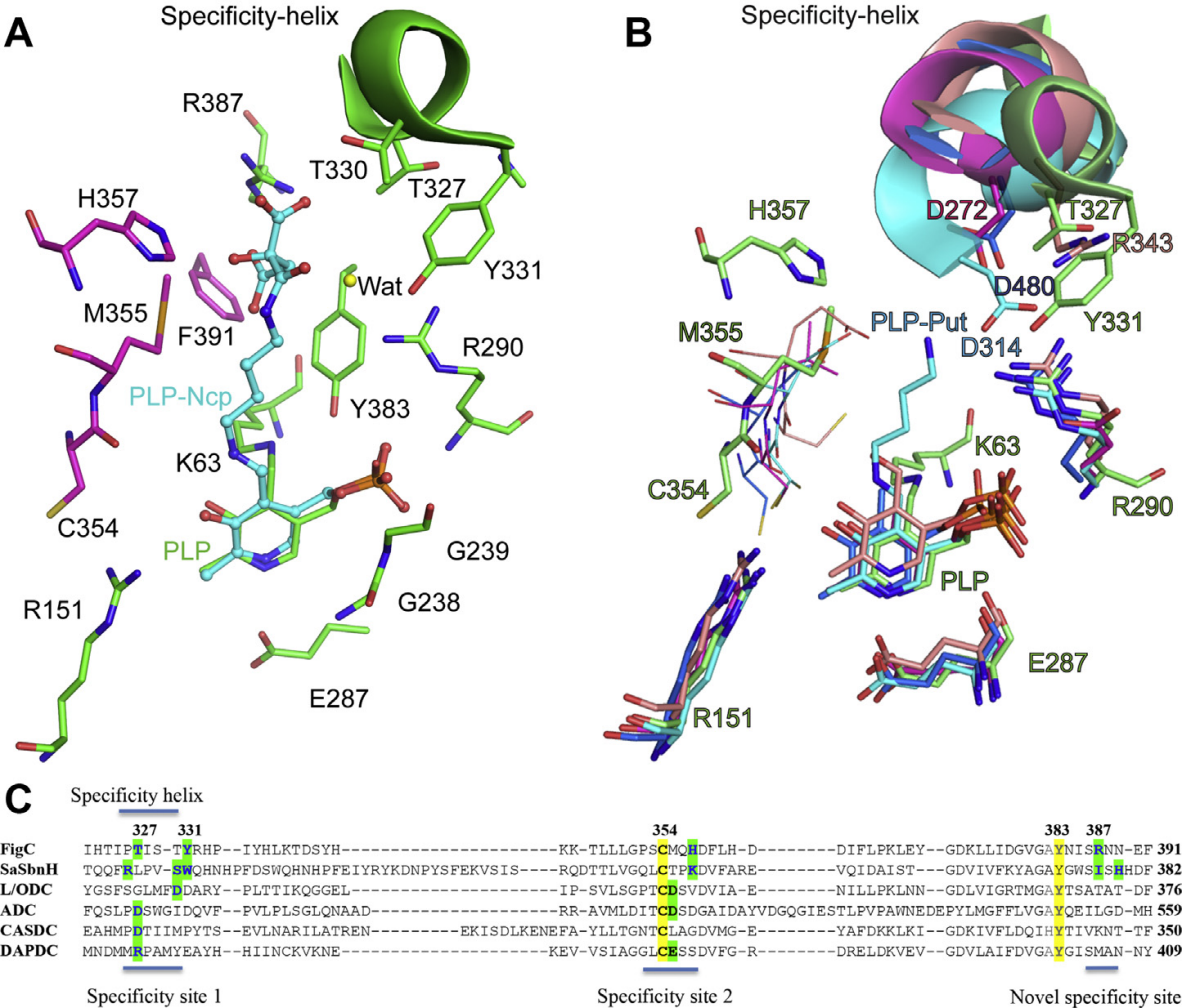
**Specificity determinants of representative PLP-dependent enzymes from the β/α structural fold.***A*, active site figure of FigC overlaid with a docked ligand (PLP-*N*-citrylputrescine [PLP-Ncp]). Subunit (a) is colored in *pink* and subunit (b) is colored in *green*. The structurally determined position for PLP is shown in *green* bound covalently to K63. The docked ligand PLP-Ncp is shown in *turquoise* as ball and sticks. A potentially relevant active site water molecule (Wat) is displayed as a *yellow sphere*. *B*, structural alignment of FigC with representative PLP-dependent enzymes from the β/α structural fold. FigC is displayed in *green*; *M. jannaschii meso*-diaminopimelate decarboxylase (1TWI) is displayed in *salmon*; *Campylobacter jejuni* carboxynorspermidine decarboxylase (3N29) is *magenta*; *Vibrio vulnificus* lysine/ornithine decarboxylase in complex with putrescine (2PLJ) is *turquoise*, and *V. vulnificus* arginine decarboxylase (3N2O) is *dark blue*. Residues are labeled for the FigC structure (shown in *green*), and additional residues from the other structures are also labeled using the above color scheme. The specificity helix is shown as a cartoon. *C*, sequence alignment of representative PLP-dependent enzymes from the β/α structural fold. The alignment was generated based on the structural alignment and shows the region of the sequence most relevant to substrate specificity. ADC, arginine decarboxylase; CASDC, L-carboxyspermidine decarboxylase; DAPDC, *meso*-diaminopimelate decarboxylase; L-ODC, L-ornithine decarboxylase.

**Figure 8:**
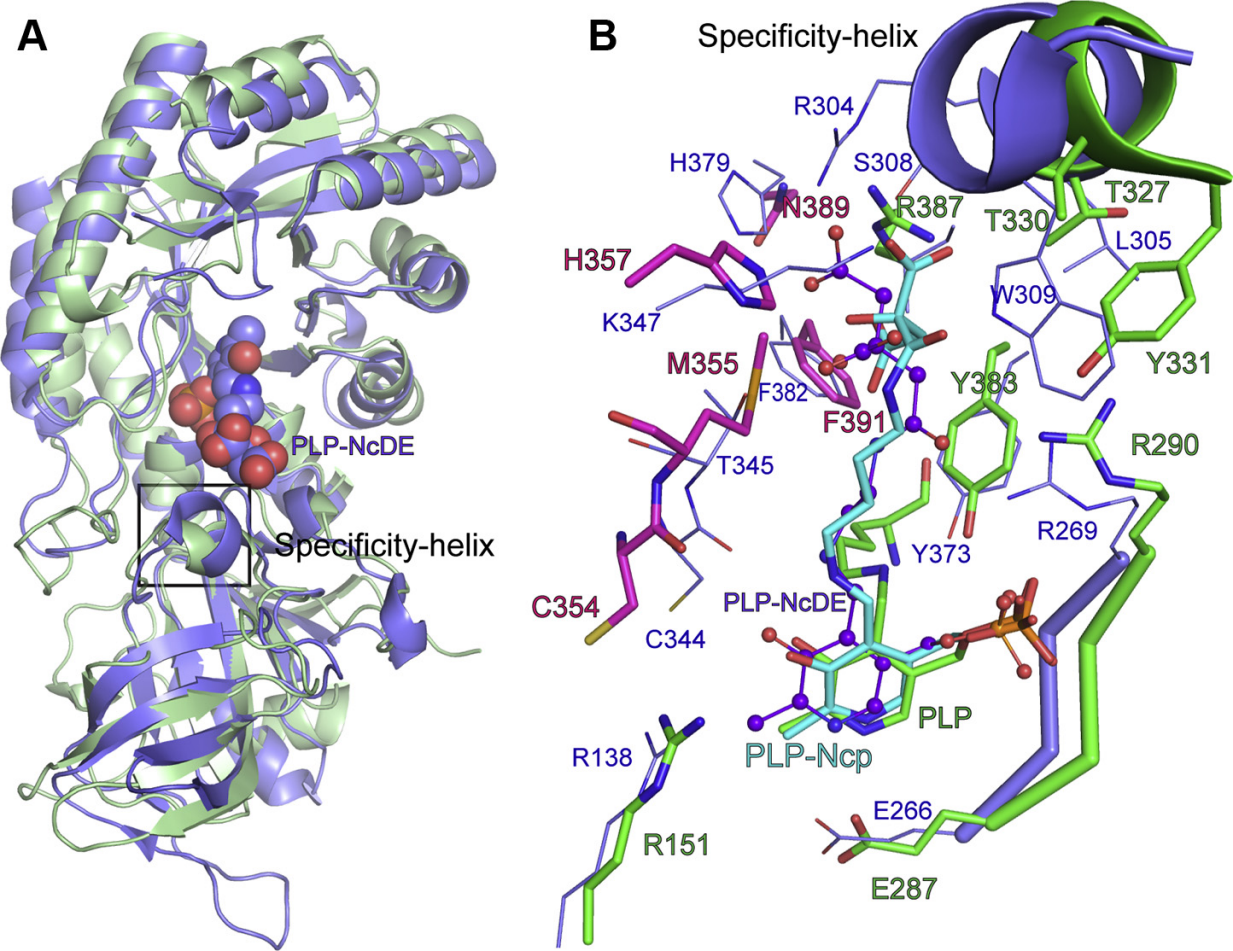
**Comparison of the substrate binding sites between FigC and SbnH.***A*, ribbon diagram showing an alignment of FigC (*pink* and *green* as in Fig. 7) with SbnH (pdb 6knk) (*purple*). Structures were aligned in Pymol using the align command to an RMSD of 2.7 Å over 3211 atoms. *N*-citryl-2,3-diaminoethane (NcDE) bound to SbnH is displayed as large spheres. The PLP bound to FigC is not shown. *B*, alignment of the active site of FigC overlaid with a docked ligand (PLP-*N*-citrylputrescine [PLP-Ncp]) (*green*) with SbnH (*purple*). PLP-Ncp is shown in turquoise as sticks, and PLP-NcDE is shown in *purple* as ball and sticks. The SbnH structures is displayed in thinner sticks, and the font sized used to label the SbnH residues is smaller than for FigC for clarity.

*N*-citrylputrescine and *N*-citrylornithine are not commercially available; therefore, we used molecular docking approaches to dock PLP-*N*-citrylputrescine into the FigC active site, thereby providing insight into the nature of the substrate-binding site. We chose to dock the product in its imine form with the PLP cofactor so that the position of the docked PLP could be used to provide an anchor for the docked pose. We restricted the PLP pocket so that residues could not move, and we restricted the PLP conformation to the crystallographic structure for the docking run. The top scoring docked pose shows the ligand in an extended conformation bound in the dimer interface (Fig. 7*A* and Table S2), and the substrate-binding pocket that is defined by this model overlaps with the binding pocket that has been observed for other members of this enzyme family, as illustrated by putrescine in the solved structure of the *V. vulnificus* L/ODC (45) (Fig. 7*B*), and for *Staphylococcus aureus* SbnH, an *N*-citryl-L-2,3-diaminopropionic acid decarboxylase (46), bound to *N*-citryl-2,3-diaminoethane (Fig. 8).

The PLP-*N*-citrylputrescine/FigC model predicts that the ligand will form key interactions with H357 and R387, with H537 predicted to H-bond with the ligand hydroxyl (O2) and R387 in position to form a salt-bridge with both citrate carboxyl groups (R387 NE to O3 and NH1 to O5 of the ligand). R290 and Y331, which project from the 3_10_-helix at the back of the pocket, are predicted to form an H-bond network with the oxygen (O1) of the peptide bond through a bound water, provided that the observed bound water would also be present in a ligand-bound structure. The aliphatic portion of the docked ligand is predicted to stack against Y383 and M355. Two threonine residues T330 and T327 projecting from the 3_10_-helix may also contribute to substrate interactions, though in the case of T327, the rotamer observed in the unliganded crystal structure places the methyl group near the ligand. This suggests that the opposite rotamer might be present in a ligand-bound structure to allow the β-hydroxyl to be in position to form H-bonds with the imine nitrogen of the PLP-substrate.

### Structural basis for the FigC substrate specificity

Prior structures of enzymes from AR-fold PLP-dependent decarboxylases have uncovered several key principles that explain how this enzyme family has evolved to catalyze decarboxylation of such a diverse group of amino acid substrates (45, 47). In structures with bound product or substrate analogs, the substrate binds at the dimer interface forming interactions with the C354 loop in the C-terminal domain and with a 3_10_-helix in the N-terminal domain, that we have previously named the specificity helix, and which is positioned at the back of the pocket (Fig. 7*B*). Both the amino acid composition and the position of the C354 loop and the 3_10_-helix are variable, thereby providing a mechanism to modify the active site to accommodate different ligands of variable shape, chemistry, and size.

Alignment of the FigC active site structure with those from other enzymes in the family (DAPDC, CASDC, L/ODC, ADC, and SbnH) was performed to provide insight into the structural basis for the novel *N*-citrylornithine ligand specificity (Fig. 7*C*). Clear differences emerge in the positioning of the 3_10_-helix, the amino acid residues that project from it, and in the amino acids present on the C354 loop. Owing to the larger size of the *N*-citrylornithine substrate, novel interactions may be formed that involve residues positioned further back in the pocket compared with other ligands. First, the 3_10_-helix has rotated out of the pocket to a greater extent than for any of the other structural homologs, and it projects two threonine residues (T327 and T330) and a tyrosine (Y331) into the pocket (Fig. 7, *A*–*B*), all of which are likely to be in position to interact with the substrate based on the docked structure. These residues replace the negatively charged aspartate residues that are involved in binding of the basic amino acid substrates ornithine (D314 in L/ODC), arginine (D480 in ADC), carboxyspermidine (D272 in CASDC), and the DAPDC arginine residue (R343) that interacts with the carboxylate of *meso*-diaminopimelate. Second, there are considerable differences in the role of residues in the C354 loop with respect to potential ligand interactions. The aspartate positioned next to the catalytic cysteine in L/ODC, ADC, and DAPDC, which interacts with the substrate side-chain amino group has been replaced by M355 in FigC. The modeled ligand structure in FigC suggests that H357, which is positioned deeper into the pocket will play an analogous role in FigC to the missing aspartate and will interact with the ligand hydroxyl and potentially one of the citryl carboxylate groups. Finally, as noted above, the FigC structure has also evolved to use additional structural elements that are deeper in the pocket for substrate binding (*e.g.*, R387), elements that were not needed for binding to the smaller substrates preferred by other enzymes in the family.

A comparator structure with a similarly sized ligand is the SbnH structure bound to *N*-citryl-2,3-diaminoethane, which is similar to *N*-citrylputrescine in containing two carboxylates and a hydroxyl on the ligand chain, but which has a linker region between the two amino groups that is two carbons shorter (Fig. 8). This structure confirms our prediction of the relative position of *N*-citrylputrescine in the binding pocket. In the case of the SbnH structure, it has replaced the H357 in FigC with K347, which forms a salt-bridge with one citryl carboxylate (back facing), and it projects a novel basic residue (R304) and S308 from the back of the 3_10_-specificity helix, with both forming H-bonds with the other carboxylate (back facing). Residue W309 in SbnH is also projected from the 3_10_-helix and takes the place of Y331 in FigC, though it forms a salt-bridge with the same carboxylate as K347. In the SbnH structure H379 is within 4.2 Å of the back-facing carboxylate, and this residue is N389 in FigC, suggesting the possibility that if the ligand adopted a less kinked structure than predicted that N389 could be involved with binding interactions with substrate for FigC as well. Indeed, one important consideration of the docked structure is that while in the apostructure, the 3_10_-helix loop is extended to open the pocket compared with enzymes that recognize shorter ligands, and it remains possible that the loop is dynamic and may assume a different position upon substrate binding. For example, it may move toward the ligand, which might allow the substrate to bind in a more elongated pose that would better position both R387 and N389 to provide stabilizing contacts with the citrate carboxyl groups.

### FigC belongs to a subfamily of alanine racemase-fold decarboxylases unrelated to polyamine metabolism

**Figure 9:**
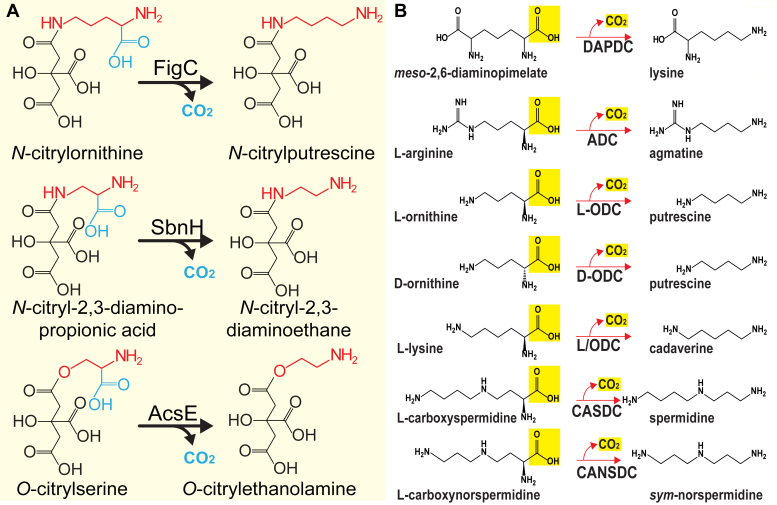
**Alanine racemase-fold PLP-dependent decarboxylase reactions.***A*, citrylamino acid decarboxylases: FigC, *N*-citryl-L-ornithine decarboxylase; SbnH, *N*-citryl-L-2,3-diaminopropionic acid decarboxylase; AcsE, *O*-citryl-L-serine decarboxylase. *B*, other AR-fold PLP-dependent decarboxylases: DAPDC, *meso*-diaminopimelate decarboxylase; ADC, L-arginine decarboxylase; L-ODC, L-ornithine decarboxylase; D-ODC, D-ornithine decarboxylase; L/ODC, bifunctional L-lysine/L-ornithine decarboxylase; CASDC, L-carboxyspermidine decarboxylase; CANSDC, L-carboxynorspermidine decarboxylase.

The FigC protein exhibits homology to two other siderophore biosynthetic enzymes that are not involved in polyamine metabolism. The *S. aureus* SbnH PLP-dependent decarboxylase (48) is involved in the biosynthesis of staphyloferrin B and decarboxylates *N*-citryl-2,3-diaminopropionic acid to form *N*-citryl-2,3-diaminoethane (Fig. 9*A*). Achromobactin biosynthesis in *Pseudomonas syringae* includes a PLP-dependent decarboxylase AcsE (49) that decarboxylates *O*-citrylserine to form *O*-citrylethanolamine (Fig. 9*A*). In addition to the three decarboxylases involved in siderophore biosynthesis, FigC, SbnH, and AcsE, there are two other known homologous AR-fold decarboxylases that are not involved in polyamine metabolism. The *E. coli* peptidyl-nucleotide translation inhibitor microcin C (McC) is a heptapeptide containing an aspartate α-carboxyl group linked to AMP. During the maturation of McC, a 3-amino-3-carboxypropyl group is transferred to the McC phosphate group, and the final stage of maturation involves the decarboxylation of the 3-amino-3-carboxypropyl–modified McC intermediate by the AR-fold decarboxylase MccE to form the mature microcin C (50). Finally, BtrK is a PLP-dependent AR-fold decarboxylase involved in the biosynthesis of the butirosin aminoglycoside antibiotics of *Bacillus circulans*. The BtrK enzyme decarboxylates BtrI-*S*-glutamate to form BtrI-*S*-γ-aminobutyric acid, where BtrI is an 87 amino acid carrier protein (51).

**Figure 10:**
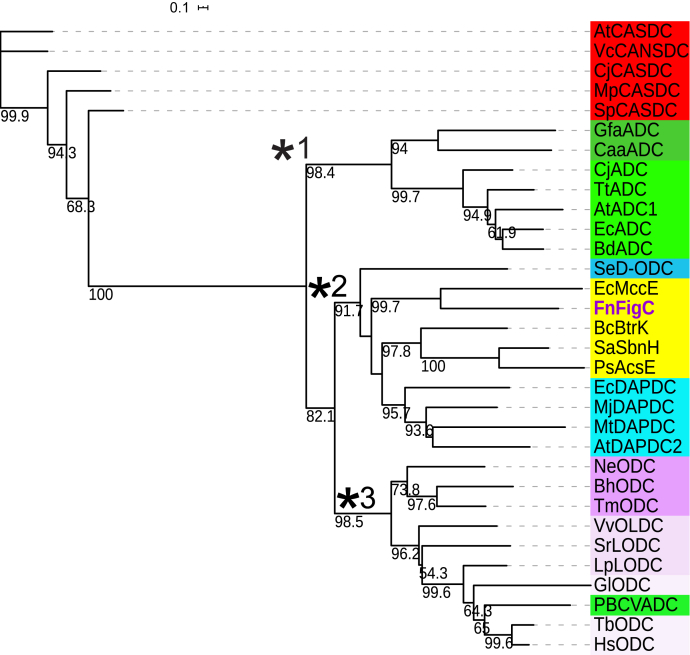
**Unrooted maximum likelihood tree of the alanine racemase-fold PLP-dependent decarboxylases.** AtCASDC, *Agrobacterium fabrum* str. C58 (α-Proteobacteria) CASDC (NP_356481); VcCANSDC, *Vibrio cholerae* (γ-Proteobacteria) CANSDC (C6YCI2); CjCASDC, *Campylobacter jejuni* (ε-Proteobacteria) CASDC (WP_002877469); MpCASDC, *Methanolacinia paynteri* (Archaea, Euryarchaeota) CASDC (WP_048150085); GfaADC, *Gramella forsetii* (Bacteroidetes) ancestral short-form ADC (YP_863630); CaaADC, *Chloroflexus aurantiacus* (Chloroflexi) ancestral short-form ADC (YP_001634722); CjADC, *Campylobacter jejuni* (ε-Proteobacteria) long-form ADC (WP_002871443); TtADC, *Thermus thermophilus* (Deinococcus-Thermus) long-form ADC (AAS81619); AtADC1, *Arabidopsis thaliana* (Eukaryota, Viridiplantae) long-form ADC (AAB09723); EcADC, *Escherichia coli* (γ-Proteobacteria) long-form ADC (AAA24646); BdADC, *Bacteroides dorei* (Bacteroidetes) long-form ADC (AII67066); SeD-ODC, *Salmonella enterica* subsp. *enterica* serovar Typhimurium (γ-Proteobacteria) D-ODC (AAL21261); EcMccE, *E. coli* (γ-Proteobacteria) microcin C7 protein MccE (YP_006953769); FnFigC, *Francisella tularensis* subsp. *novicida* U112 (γ-Proteobacteria) citrylornithine decarboxylase FigC (ABF50970); BcBtrK, *Bacillus circulans* (Firmicutes) butirosin biosynthesis protein K (Q2L4H3); SaSbnH, *Staphylococcus aureus* (Firmicutes) citryl-L-2,3-diaminopropanoate decarboxylase (AAP82070); PsAcsE, *Pseudomonas syringae* (γ-Proteobacteria) *O*-citryl-L-serine decarboxylase (ELS42881); EcDAPDC, *E. coli* (γ-Proteobacteria) DAPDC (NP_417315); MjDAPDC, *Methanocaldococcus jannaschii* (archaea, euryarchaeota) DAPDC (Q58497); MtDAPDC, *Mycobacterium tuberculosis* (Actinobacteria) DAPDC (2O0T); AtDAPDC2, *Arabidopsis thaliana* (Eukaryota, Viridiplantae) DAPDC (Q94A94); NeODC, *Nitrosomonas europaea* (β-Proteobacteria) L-ODC (WP_011111520);BhODC, *Bartonella henselae*, (α-Proteobacteria) L-ODC (WP_011181090); TmODC, *Thermotoga maritima* (Thermotogae) L-ODC (NP_229669); VvOLDC, *Vibrio vulnificus* (γ-Proteobacteria) L-ornithine/L-lysine decarboxylase (AAO07938); SrLODC, *Selenomonas ruminantium* (Firmicutes) L-lysine/L-ornithine decarboxylase (BAA24923); LpLODC, *Lupinus angustifolius* (Eukaryota, Viridiplantae) L-lysine/L-ornithine decarboxylase (BAK32797); GlODC, *Giardia lamblia* (Eukaryota, Excavata) L-ornithine decarboxylase (EFO63849); PBCVADC, *Paramecium bursaria* chlorella virus 1 (chlorovirus) L-ODC-like ADC (NP_048554); TbODC, *Trypanosoma brucei* (Eukaryota, Excavata) L-ODC (AAA30218); HsODC, *Homo sapiens* (Eukaryota, Opisthokonta) L-ODC (AAA59967). The approximately 80 amino acid insertion in the long form ADCs relative to the other decarboxylases was removed, and all N termini and C termini sequences were trimmed to facilitate the alignment. ADC, arginine decarboxylase; CANSDC, L-carboxynorspermidine decarboxylase; CASDC, L-carboxyspermidine decarboxylase; DAPDC, *meso*-diaminopimelate decarboxylase; D-ODC, D-ornithine decarboxylase; L-ODC, L-ornithine decarboxylase.

Polyamine-related AR-fold decarboxylases consist of the ancestral ADCs (which are of a similar size to DAPDC and L-ODC), the derived ADCs, which are larger because of an internal insertion and C-terminal extension, the L-ODC, D-ornithine decarboxylase (D-ODC), and bifunctional L/ODC, the ODC homolog of *Paramecium bursaria Chlorella virus* that has become repurposed to be an ADC, CASDC, and carboxynorspermidine decarboxylase (CANSDC). In addition, DAPDC is involved in the final step of lysine biosynthesis (47, 52, 53). The substrates and products of these enzymes are shown in Fig. 9*B*. When these AR-fold decarboxylases are aligned (after trimming of N termini and C termini and removal of the internal insertion of the derived ADC), an unrooted maximum likelihood tree indicates that the FigC, SbnH, AcsD, MccE, and BtrK proteins form a strongly supported clade (clade 2, Fig. 10) together with bacterial and plant DAPDCs and the D-ODC. A separate strongly supported clade (clade 3) consists of bacterial and eukaryotic ODCs, bacterial and plant bifunctional L/ODCs, and the repurposed *Paramecium bursaria Chlorella virus*-1 ADC. Ancestral and derived ADCs form a third strongly supported clade (clade 1), and the CASDCs and CANSDCs are excluded from the other clades. The ML tree indicates that the *N*-citrylornithine decarboxylase FigC and the other polyamine-unrelated decarboxylases (SbnH, AcsE, MccE, and BtrK) share a common ancestor with DAPDC, rather than with L-ornithine decarboxylase (Fig. 10).

## Discussion

### Putrescine prototrophy and putrescine-dependent rhizoferrin biosynthesis

Two bacterial polycarboxylate siderophores are close structural analogs of rhizoferrin, and each contains two citrate molecules bridged by an amino acid. One is the *S. aureus* siderophore staphyloferrin A, containing D-ornithine (54), the other is the L-lysine-containing cornyebactin, produced by *Clostridium diphtheriae* (55). Corynebactin is likely to be made by the simpler biosynthetic pathway, consisting of a single large protein CiuE composed of two fused NIS synthetase domains. Staphyloferrin A is synthesized by two separate NIS synthetases and a PLP-dependent amino acid racemase (56). Both of these biosynthetic pathways are more complex and therefore more expensive than the *R. pickettii* rhizoferrin pathway. It is probable that two different NIS synthetase components are required for corynebactin and staphyloferrin A biosynthesis because of the unsymmetrical structure of the amino acid component. Why might *S. aureus* and *C. diphtheriae* use the more complex amino acid–containing structures rather than a putrescine-containing one? It is well known that *S. aureus* is a putrescine auxotroph (57, 58), and BLASTP analysis of *C. diphtheriae* strain proteomes indicates that no known putrescine synthesizing enzymes (52) are encoded in their genomes, in contrast to the putrescine prototrophs *R. pickettii* (59) and *L. pneumophila*.

Rhizoferrin biosynthesis in the fungus *R. delemar* also requires only one NIS synthetase, but the fungal and bacterial rhizoferrin molecules are different structural enantiomers. The fungal and *R. pickettii* rhizoferrin-producing NIS synthetases also differ in other respects. Fungal rhizoferrin NIS synthetase has an amine preference of 1,3-diaminopropane > putrescine > cadaverine > L-ornithine, as measured *in vitro* by consumption of NADH (20). The *R. pickettii* enzyme examined here appears to exhibit similar preference for putrescine and cadaverine, with a lower preference for 1,3-diaminopropane and no activity with L-ornithine, as detected *in vitro* using the CAS reagent. Only a monosubstituted *N*-citrylcadaverine is produced by the fungal enzyme *in vitro* with cadaverine, whereas the disubstituted cadaverine analog homorhizoferrin is produced *in vitro* with the *R. pickettii* enzyme. However, *R. pickettii* does not produce cadaverine (59), so it is unlikely that there could be native biosynthesis of homorhizoferrin. Indeed, only rhizoferrin but not homorhizoferrin has been detected in *R. pickettii* (21).

The fungal and *R. pickettii* NIS synthetases produced rhizoferrin when expressed heterologously in *E. coli*. Carroll *et al.* (20) grew an *E. coli* strain expressing the fungal enzyme on solid medium supplemented with 1 mM citrate and putrescine, presumably with the aim of increasing rhizoferrin production. This is unlikely to increase rhizoferrin synthesis because *E. coli* is incapable of taking up citrate under aerobic conditions (60). Biotechnological optimization of rhizoferrin production in *E. coli* will therefore need to incorporate co-expression of an aerobically expressible citrate uptake transporter or possibly another citrate synthase, whereas putrescine is natively abundant in *E. coli*.

### Polyamine auxotrophy and putrescine-circumventing rhizoferrin biosynthesis

The clearest sign that rhizoferrin is produced by a different pathway in *Francisella* is that, besides the NIS synthetase FslA/FigA, a gene encoding a PLP-dependent decarboxylase (FslC/FigC) homologous to ODC and DAPDC is also essential for rhizoferrin biosynthesis (34). We have shown here that the NIS synthetase FigA cannot produce rhizoferrin in *E. coli*, but co-expression of FigA and FigC enables rhizoferrin production. Recently, it has been shown that a *figC* deletion mutant of the *F. tularensis* LVS strain accumulates monosubstituted *N*-citrylornithine (35), and here, we have corroborated this finding with the expression of *F. novicida figA* in *E. coli*, which heterologously produces monosubstituted *N*-citrylornithine. Unlike in *R. pickettii* and *L. pneumophila* where citrate is directly conjugated with putrescine, *F. tularensis* and *F. novicida* must conjugate citrate first with L-ornithine and then decarboxylate the *N*-citrylornithine to form *N*-citrylputrescine. Using purified recombinant *F. novicida* FigA and FigC enzymes with *in vitro* assays, we found that neither D-ornithine, 1,3-diaminopropane, putrescine, cadaverine, L-lysine, and L-2,4-diaminobutyrate nor L-2,3-diaminopropionate can form a functional siderophore, and only L-ornithine is able to do this. This substrate specificity is narrower than the rhizoferrin NIS synthetase from *R. pickettii*, but we do not know formally which step, FigA, FigC, or both, is the substrate gatekeeper. However, as putrescine, cadaverine, and 1,3-diaminopropane do not require decarboxylation, and we know that the recombinant *R. pickettii* NIS synthetase can produce strong (homorhizoferrin) and weak (norrhizoferrin) siderophore activity *in vitro* with these amines, and it would suggest that the *F. novicida* FigA is the substrate gatekeeper, at least for amines, otherwise the *F. novicida* FigA synthetase would produce rhizoferrin, homorhizoferrin, and norrhizoferrin.

What might explain why *R. pickettii* and *L. pneumophila* produce rhizoferrin from putrescine, but *F. tularensis* and *F. novicida* must produce it from L-ornithine, requiring an additional *N*-citrylornithine decarboxylase? Putrescine is synthesized in *R. pickettii* and *L. pneumophila*, although by completely different pathways through convergent evolution. Not only are *F. tularensis* and *F. novicida* putrescine auxotrophs due to genomic decay of the arginine decarboxylase, agmatine deiminase, and *N*-carbamoylputrescine amidohydrolase encoding genes, they are also arginine and ornithine auxotrophs (61). Although *Francisella* replicates in the host cytoplasm where ornithine and putrescine can be accessed, only L-ornithine is incorporated into rhizoferrin. Spermine or spermidine from the host are essential for optimal growth of *Francisella*, whereas putrescine is considerably less effective (62). It is likely that putrescine would compete for uptake with spermine or spermidine, unlike ornithine, and would inhibit uptake of spermidine/spermine that is required for optimal growth. The *E. coli* PotABCD polyamine transporter takes up putrescine and spermidine (and spermine) but prefers spermidine (63). In the light of this polyamine uptake competition, the *N*-citrylornithine pathway could be viewed as a putrescine circumvention mechanism. This is reminiscent of the aminopropylagmatine pathway of spermidine biosynthesis in *Thermus thermophilus* and *Thermococcus kodakarensis*, which has replaced the usual aminopropylputrescine pathway (64, 65). Unlike in most organisms that synthesize spermidine from putrescine, the aminopropylagmatine pathway circumvents a putrescine requirement for spermidine biosynthesis.

### An expandable and mutable active site to accommodate diverse substrates

The FigC PLP-dependent *N*-citrylornithine decarboxylase is related to the AR-fold ODCs and DAPDCs. To understand how FigC is able to decarboxylate the larger *N*-citrylornithine substrate, we determined its X-ray crystal structure. Not surprisingly, the FigC monomer exhibits the typical N-terminal β/α-barrel domain and C-terminal β-barrel domain typical of this family. Like other family members, the active site is located at the interface of the monomer domains and between the N-terminal domain of one dimer subunit and the C-terminal domain of the other. However, compared with ODC, L/ODC, ADC, DAPDC, and CASDC, clear differences can be discerned in FigC. The modeled *N*-citrylputrescine ligand indicates that the FigC *N*-citrylornithine substrate interacts with residues further back in the active site binding pocket, the 3_10_-helix that constitutes the specificity helix in the N-terminal domain has rotated out of the binding pocket to accommodate the larger substrate, and amino acid replacements allow interaction with the citrate moiety. There are also changes to the C354 loop that provide substrate specificity, the cysteine in this position, which is conserved in all PLP-dependent AR-fold decarboxylases, functions as a catalytic base in ODC. The AR-fold decarboxylase family possesses the characteristics of a successful enzyme family: a robust structural chassis, adjustable active site size and changing amino acid composition for interacting with different substrates.

### A subfamily of polyamine-unrelated AR-fold PLP-dependent decarboxylases

Phylogenetic analysis places the FigC *N*-citrylornithine decarboxylase in a robustly supported clade (clade 2) with SbnH and AcsE that decarboxylate *N*-citryl-2,3-diaminopropionate and *O*-citrylserine, respectively, and with MccE and BtrK, and DAPDCs and D-ODC. The structure of SbnH has been solved recently and is similar to the other enzymes in this family (46), and its activity is inhibited by free citrate. MccE decarboxylates a translation inhibitor microcin C intermediate that has been modified with a 3-amino-3-carboxypropyl group that then becomes an aminopropyl modification after decarboxylation by MccE (50). The BtrK enzyme decarboxylates an L-glutamylated, 87 amino acid carrier protein BtrI to produce γ-aminobutyrate-S-BtrI (51). DAPDC is the final step in lysine biosynthesis, and D-ODC, although it converts D-ornithine into putrescine, is unlikely to be involved in polyamine (spermidine or homospermidine) metabolism (66, 67). This clade of enzymes is characterized by an absence of any polyamine biosynthesis-related role, unlike the other enzymes of this family (clades 1 & 3, and CASDC/CANSDC). Any PLP-dependent AR-fold decarboxylase homolog that falls into clade 2 after phylogenetic analysis is unlikely to be involved in polyamine metabolism and is more likely, if not a DAPDC or D-ODC, to be involved in biosynthesis of specialized metabolites, such as siderophores.

## Experimental procedures

### Bacterial strains and growth conditions

Strains of *E. coli* were grown in LB (Luria broth) or in M9 salts minimal medium. Selection for the pETDuet-1 plasmid (Novagen) in *E. coli* BL21 (Novagen) was 100 μg/ml ampicillin and for pACYCDuet-1, 34 μg/ml chloramphenicol.

### Recombinant DNA methods

Plasmid DNA was purified using PureYield Plasmid Miniprep System (Promega) according to the manufacturer’s protocol. Polymerase chain reactions were performed using Phusion High-Fidelity DNA Polymerases (New England Biolabs). The following open reading frames were synthesized with *E. coli*-optimized codons by GenScript Corp.: *R. pickettii* 12D NIS synthetase (GenBank, WP_012761004); *R. pickettii* MFS efflux transporter (WP_012761003); *F. novicida* U112 NIS synthetase (FigA, WP_003041317); *F. novicida* U112 MFS efflux transporter (FigB, WP_003041320); *F. novicida* U112 AR-fold PLP-dependent decarboxylase (FigC, WP_003041325); *L. pneumophila* subsp. pneumophila str. Philadelphia 1 NIS synthetase (WP_010947056); the first (N-terminal) 19 amino acids were subsequently removed. Synthesized genes were subcloned into the NdeI and XhoI (for synthetases) sites or NcoI and EcoRI (for transporters) of pETDuet-1 or pACYCDuet-1 plasmids (Novagen). Plasmid constructs were verified by sequencing and then transformed into *E. coli* BL21 competent cells. Strains-containing plasmid constructs were grown in LB or M9 salts minimal medium supplemented with the appropriate antibiotic at 30 °C until an OD_600_ of 0.5 and induced with 0.5 mM isopropyl-β-D-1-thiogalactopyranoside (IPTG). The culture was further incubated at 30 °C overnight before collecting for processing. For the production of purified recombinant *F. novicida* FigA and FigC proteins and the *R. pickettii* NIS synthetase protein, the ORFs were cloned into pET28b-TEV using 5’-BamH1 and 3’-HindIII sites. For expression of plasmid pETDuet-1 containing FigA + FigC, 10 μM FeCl3.6H2O was added to the M9 salts minimal medium.

### Protein overexpression and purification for *in vitro* assays

*F. novicida figA* and *figC* were each expressed from pET28b containing a TEV protease site after the his-tag (MGHHHHHHAENLYFQGADP), in *E. coli* BL21(DE3). Cells were grown to mid-log phase at 37 °C in LB with aeration, followed by addition of IPTG at 0.2 mM and further culture of the cells overnight at 16 °C. Cells were then pelleted and resuspended in 50 mM HEPES buffer (pH 7.4), 500 mM NaCl, 10 mM imidazole, and lysed in a cell disruptor at 10,000 psi. The lysate was centrifuged to remove unbroken cells, debris, and insoluble material. Soluble sample was applied to a 5 ml Hi-Trap chelating HP column (GE Healthcare) equilibrated with NiSO4 and buffer A, and the 6xHis-tagged proteins were eluted from the column with a gradient of 0 to 80% buffer B over 20 column volumes. Buffer A contained 50 mM HEPES (pH 7.5) 500 mM NaCl, 10 mM imidazole, and buffer B contained 50 mM HEPES (pH 7.5), 500 mm NaCl, and 500 mM imidazole. Proteins were desalted into 10 mM Tris-HCl (pH 7.5), 150 mM NaCl, and 10% glycerol at 4 °C using a Hi-prep 26/10 desalting column (GE Healthcare). Protein purity was confirmed by using SDS-polyacrylamide gel electrophoresis. Protein concentration was determined using a Biotek Synergy Multi-Mode Microplate reader at OD_280_ read with a molecular weight and protein extinction coefficient program. Yield of protein from the induced cultures harboring the expression constructs was determined to be 8.8 mg/L (FigA), 72.4 mg/L (FigC), and 4.8 mg/L (*R. pickettii* NIS synthetase).

### Siderophore production assays

The iron scavenging activity of bacterial plate-grown colonies or liquid culture supernatants was detected with CAS agar plates or liquid solution (68). Cells of *E. coli* BL21 expressing the siderophore synthetases and/or transporters were grown overnight in LB medium. After adjusting cell density at OD_600_ to 1.0, a 3 μl aliquot was spotted onto CAS LB agar plates and allowed to dry before incubation at 37 °C. Orange halos indicated positive iron-scavenging activity.

### Siderophore partial purification

Liquid cultures of *E. coli* BL21 were grown for 20 h in LB medium at 250 rpm, 30 °C after induction with 0.5 mM IPTG when the cells had reached approximately 0.5 OD_600_. Cells were then pelleted at 4 °C. Culture supernatant was passed through a Dowex 50WX8 column (H form), and the flowthrough was adjusted to pH 7.0 with NaOH. The sample was then applied to an AGI1-X8 column (formate form, Bio-Rad) that was then washed with 5 volumes of water and eluted with 3 volumes of 40% formic acid. The eluate was vacuum dried at 35 °C and reconstituted with 1/20 of the original volume, and the solution was neutralized with NaOH. Using a 3.0 kDa cutoff Amicon concentration filter, the resulting filtrate was dialysized to remove salt before carrying out mass spectrometric analysis. Sufficient siderophore remained after filtration to allow identification by the CAS reagent and mass spectrometry. Siderophore-containing fractions were detected by CAS liquid assay throughout the procedure. The partially purified siderophore was analyzed using an Agilent 1100 series LC-MS equipped with a Waters 2487 dual-wavelength absorbance detector (210 and 254 nm), column oven (30 °C), and an Agilent Eclipse XDB-C18 column (4.6 × 150 mm, 5 μm) couple to a Waters Quattro Micro API (atmospheric pressure ionization) mass spectrometer in positive mode with a scan range 100 to 640 m/z. MassLynx software (Waters) was used for data acquisition. A yellowish pellet of the partially purified siderophore was dissolved in 0.5 ml of 50% methanol and filtered. The LC was carried out at a flow rate of 0.5 ml/min with a 50 μl injection volume, using an isocratic elution starting with 20% aqueous acetonitrile containing 0.1% formic acid and with a hold at 50% over 10 min followed by a hold at 90% for a further 10 min. For the MS component, the cone voltage and desolvation temperature was 50V and 150 °C, respectively.

### *In vitro* siderophore biosynthesis and detection

For the *F. novicida* FigA and FigC proteins, reactions were performed in 200 μl total volume containing 2.25 mM ATP, 15 mM MgCl_2_, 3 mM Na-citrate, 10 mM L-ornithine, 20 μM PLP, and 50 mM HEPES pH 7.4. FigA and FigC were present at 2 μM. The *R. pickettii* NIS synthetase reaction used the same reaction components as FigA and FigC except 10 mM putrescine replaced L-ornithine, PLP was not included, and the NIS synthetase was present at 1.5 μM. Alternative substrates were tested at 10 mM. Reactions were incubated for 2 h at RT, in the dark. After the reaction, enzymes were inactivated by heating to 70 °C for 15 min, followed by centrifugation at 14,800*g* for 20 min to remove precipitate. An equal volume of CAS solution was added to the supernatant to detect siderophore activity. For LC-MS analysis, the reactions were performed as described above except the supernatants of 10 parallel reactions were pooled (total 2.0 ml) and evaporated at 40 °C to give a final volume of 50 μl that was then analyzed by LC-MS as described above for the partially purified siderophore. Pure polyamines and Na-citrate were obtained from Sigma Aldrich.

### *F. novicida* FigC protein purification for crystallization

*E.coli* BL21 (Novagen) was used to express *figC*, and protein was purified sequentially with nickel IMAC and gel-filtration chromatography. Briefly, cells were grown to 0.8 OD_600_ at 37 °C, 0.2 mM IPTG was added to induce protein expression, and cells were grown overnight at 16 °C. Cells were pelleted by centrifugation (4000*g*), and the pellet resuspended in lysis buffer (100 mM HEPES pH 8.0, 50 mM NaCl, 5 mM imidazole, 20 μM PLP and 0.02% Brij 35 detergent) (Affymetrix), containing protease-inhibitor cocktail. Cells were lysed by three passes through an EmulsiFlex-C5 high pressure homogenizer (Avestin Inc), the lysate then clarified by centrifugation (20,000*g*), and the resultant supernatant was applied to a HisTrap HP column (GE Healthcare) precharged with Ni^+2^. The column was washed sequentially with lysis buffer and with lysis buffer containing 50 mM imidazole. FigC protein was then washed using a linear gradient from 50 to 300 mM imidazole, and protein was eluted around 120 mM imidazole. Fractions containing FigC were pooled, concentrated with an Amicon Ultra concentrator (Millipore), and then purified by gel filtration column chromatography on a HiLoad 16/60 Superdex 200 column (GE Healthcare) equilibrated with crystallization buffer (10 mM HEPES pH 7.8, 20 mM NaCl, 1 mM DTT). Fractions containing FigC were pooled and concentrated to 30 mg/ml. The selenomethionine FigC was expressed according the protocol provided by Molecular Dimensions using methionine auxotrophic strain T7 Express Crystal Competent *E. coli* (New England Biolab). One hundred milliliters of minimal medium (made from SelenoMet Medium Base and SelenoMet Nutrient Mix [Molecular Dimensions]) supplied with L-methionine (Molecular Dimensions) was used to grow an overnight culture at 37 °C. Cells were then pelleted and washed three times with sterile water and resuspended and inoculated into 2 L of the minimal medium supplied with L-SeMet (Molecular Dimensions) for 2 h at 37 °C. Protein expression was induced by addition of 1 mM IPTG, and cells were grown overnight at 16 °C. The selenomethioninyl-labeled protein was purified as for the native protein. All chemicals were obtained from Sigma Aldrich except when specified.

### Crystallization and data collection of FigC

Preliminary crystallization conditions were found using the crystallization conditions screen *Cryos* suite (Nextal). Conditions were then refined by variation of pH, precipitant, and protein concentrations to find optimal conditions. Crystallization was performed by hanging drop vapor diffusion at 20 °C, and the crystallization drop contained an equal volume of reservoir solution and FigC (30 mg/ml). Crystals of selenomethioninyl-substituted FigC grew at 1.7 M ammonium sulfate, 85 mM HEPES pH 7.5, 1.7% PEG_400_, 15% glycerol, and 10 mM DTT.

Diffraction data were collected at 100K on beamline 19ID at the Advanced Photon Source, and data were processed with HKL3000 (69). The crystal of selenomethioninyl-substituted FigC diffracted to 2.05 Å and displays a symmetry consistent with a space group of P2_1_2_1_2 with a cell dimension of a = 70.45, b = 279.00, c = 108.57.

### Structure determination and refinement of FigC

Crystallographic phases for FigC were determined by single-wavelength anomalous diffraction using the HKL3000 package. The structure contains four molecules of FigC in the asymmetric unit. Fifteen selenium sites were identified with SHELXC (70). After density modification and solvent flattening with DM (71), 90% of the FigC sequence was built with Buccaneer (72). The resultant model was refined against the native FigC data using Phenix (73) and re-built with COOT (74) to R_work_ and R_free_ of 0.189 and 0.211, respectively. Electron density for residues (Chain A: Met1, D170-S175; Chain B: D170-D176; Chain C: D170-I177; Chain D: Q172-N174) was missing. Thus, these residues were not built into the model. All residues were within the allowed section of the Ramachandran plot (Table S1). A total of 732 water molecules were added with ARP/wARP (75).

### Docking of PLP-bound *N*-citrylputrescine into FigC

The PLP-bound *N*-citrylputrescine ligand structure was made in ChemDraw Professional 15.1 (CambridgeSoft) and converted to the three-dimensional coordinates with Open Babel (76). Initial atomic coordinates of the PLP and putrescine moieties were identical to the crystallographic ligand bound to *Trypanosoma brucei* ornithine decarboxylase (44). Partial charges and rigid descriptors were added with AutoDock Tools (77) where all bonds in the citrate moiety and C3 to N2 of the putrescine moiety were designated as rotatable. FigC protein chains A and C were protonated, partial charges added, and heteroatoms removed in AutoDock Tools. FigC chain C Arg387 was designated as a flexible residue. AutoDock Vina (78) was used to dock the PLP-bound *N*-citrylputrescine into FigC using a minimum of 50 seeds where the active site was centered at *x* = 46.6, *y* = 23.5, *z* = 56.8 and extended 15 Å, 11 Å, and 21 Å in the *x*, *y*, and *z* axes, respectively. Docking modes were ranked based on the formation of known PLP-protein residue bonds and AutoDock Vina affinity score.

### Phylogenetic analysis

A plain text file of AR-fold PLP-dependent decarboxylase homologous protein sequences in FASTA format were aligned using MUSCLE (79) through the EMBL-EBI server (80). Sequences were manually trimmed at the N termini and C termini to facilitate alignment, and the region of the long-form arginine decarboxylase sequences corresponding to the four helical bundle interdomain insertion was manually removed. The ClustalW output file (.clw) from MUSCLE was then used to create a maximum likelihood tree in IQTREE (81), with 1000 ultrafast bootstrap analyses. To draw the maximum likelihood tree, the.treefile file obtained from the IQTREE analysis was uploaded to iTOL (82) and exported to Adobe Illustrator as an.eps file.

## Data availability

The PDB ID for the FigC *N*-citrylornithine decarboxylase structure is 7KH2. All other data are included in this article.

## Conflict of interest

All authors confirm that they have no conflict of interest with the contents of the article.
