# Supplementary material for: Alternative pathways utilize or circumvent putrescine for biosynthesis of putrescine-containing rhizoferrin
Source: J Biol Chem. 2020 Dec 10;296:100146. doi: 10.1074/jbc.RA120.016738 (PMC7857480; doi:10.1074/jbc.RA120.016738)
Supplement: Table S1 and S2 and Figures S1–S3 [file mmc1.pdf]

SUPPLEMENTARY DATA

Putrescine utilization/circumvention pathways for N^1,N^4-dicitrylputrescine (rhizoferrin) biosynthesis, and structure of *N*-citrylornithine decarboxylase

Bin Li[1], Xiaoyi Deng[1], Sok Ho Kim[1,#], Leann Burrow[1], Diane R. Tomchick[2], Margaret A. Phillips[1], Anthony J. Michael[1,*]

[1]Department of Biochemistry, University of Texas Southwestern Medical Center, Dallas, Texas, USA

[2]Department of Biophysics, University of Texas Southwestern Medical Center, Dallas, Texas, USA

[#]Current Address: Realtox Labs, 200 Business Center Dr., Reistertown, MD 21136, USA

Corresponding author: Anthony J. Michael

[*]E-mail: anthony.michael@utsouthwestern.edu

Running title: Rhizoferrin biosynthesis with or without putrescine

Table S1. FigC X-ray data collection and refinement statistics

**Data collection**	
Crystal	SeMet peak[a]
Space group	$P2_12_12$
Cell constant (Å)	*a* = 70.8, *b*=279.1, *c*=108.6
Wavelength (Å)	0.97932
Resolution range (Å)	40.65 – 2.05 (2.09 – 2.05)
Unique reflections	136495 (6761)
Multiplicity	13.2 (12.2)
Data completeness (%)	99.9 (99.5)
R_{merge} (%)[b]	7.5 (110)
R_{pim} (%)[c]	2.1 (32.2)
I/σ(I)	36.0 (2.4)
Wilson B-value ($Å^2$)	23.6
**Phase determination**	
Anomalous scatterers	selenium, 15 out of 20 possible sites (4 molecules in ASU)
Figure of merit (121.8 – 2.60 Å)	0.88
**Refinement statistics**	
Resolution range (Å)	40.65 – 2.05 (2.08 – 2.05)
No. of reflections R_{work}/R_{free}	134,364/2,065(4,824/72)
Data completeness (%)	90.9 (47.8)
Atoms (non-H protein)/water	13189/383
R_{work} / R_{free} (%)	17.37 (23.60) / 19.92 (24.63)
R.m.s.d. bond length (Å)	0.005
R.m.s.d. bond angle (°)	0.661
Mean B-value ($Å^2$)	39.0
Ramachandran plot (%) (favored/additional/disallowed)[d]	96.56/3.38/0.06
Missing residues	A: 1, 170-175; B: 1, 170-176; C: 170-177; D: 172-174

Data for the outermost shell are given in parentheses.

[a]Bijvoet-pairs were kept separate for data processing.

[b]$R_{\text{merge}} = 100\ \Sigma_h\Sigma_i|I_{h,i} - \langle I_h\rangle|/\Sigma_h\Sigma_i\ \langle I_{h,i}\rangle$, where the outer sum (h) is over the unique reflections and the inner sum (i) is over the set of independent observations of each unique reflection.

[c] $R_{\text{pim}} = 100\ \Sigma_h\Sigma_i\ [1/(n_h - 1)]^{1/2}|I_{h,i} - \langle I_h\rangle|/\Sigma_h\Sigma_i\ \langle I_{h,i}\rangle$, where n_h is the number of observations of reflections **h**.

[d]As defined by the validation suite MolProbity (Chen, V.B., Arendall, W.B.A., Headd, J.J., Keedy, D.A., Immormino, R.M., Kapral, G.J., Murray, L.W., Richardson, J.S., Richardson, D.C. (2010) *MolProbity*: all-atom structure validation for macromolecular crystallography. *Acta Cryst*. **D66**, 12-21.).

Table S2 FigC Docking Atomic Distances

Distances were calculated between hydrogen bond acceptor to donor. In cases where hydrogens were included in model refinement or with defined amine hydrogen positioning, the hydrogen to bond acceptor distance is included in parenthesis.

**Ligand**	**Protein residue**	**Protein atom**	**Ligand atom**	**Interatomic distance (Å)**	**Interaction type**
Crystallographic PLP	Glu82	OE2	PLP N1	3.3	Electrostatic/ hydrogen bond potential
	Glu287	OE1	PLP N1	2.7	Electrostatic/ hydrogen bond potential
	Gly239	N	PLP OP3	3.0 (2.1)	Hydrogen bond with Gly239 donor
	Gly289	N	PLP OP3	2.9 (2.1)	Hydrogen bond with Gly239 donor
	Arg290	N	PLP OP2	2.8 (2.0)	Hydrogen bond with Arg290 donor
	Tyr383	OH	PLP OP2	2.6 (1.8)	Hydrogen bond with Tyr383 donor
	Thr201	OH	PLP OP1	3.3 (2.4)	Hydrogen bond with Thr201 donor
Docked PLP moiety	Glu82	OE1	PLP N1	3.9	Electrostatic
	Glu287	OE2	PLP N1	3.2	Electrostatic
	Gly239	N	PLP OP4	3.1	Docked PLP suggests that Gly239 may interact with either OP3 or OP4
	Gly239	N	PLP OP3	3.5	
	Gly289	N	PLP OP2	3.2	Hydrogen bond with Gly239 donor
	Arg290	N	PLP OP1	2.9	Docked PLP suggests that Arg 290 may be too close to OP1 while hydrogen bonding with OP2
	Arg290	N	PLP OP2	2.3	
	Tyr383	OH	PLP OP2	2.7	Hydrogen bond with Tyr383 donor
	Thr201	OH	PLP OP1	3.0	Hydrogen bond with Thr201 donor is possible but docking suggests this may coordinate the citrate moiety instead
Docked putrescine moiety	None predicted				
Docked citrate moiety	Thr201	OG1	COOH 1	3.3	Hydrogen bond with Thr201 donor
	Arg290	NH2	COOH 1	3.3 (2.9)	Hydrogen bond with Arg290 donor
	Arg290	NH2	COOH 6	2.1 (1.1)	Hydrogen bond with Arg290 donor
	Thr327	OH	COOH 6	3.5	Unlikely to make hydrogen bond even with the alternate Thr conformer
	Arg387	NH1	COOH 5	3.3 (2.7)	Hydrogen bond with Arg387 donor

Figure S1 *A*, Alignment of rhizoferrin MFS efflux transporters with *E. coli* MdtG. Transporters from *F. novicida* U112 (*Fnov*FigB, YP_899294), *L. pneumophila* (*Lpne*LbtB, WP_010947055), *R. pickettii* (*Rpic*MFS, YP_002980162) and *E. coli* K12 (*Ecol*MdtG, NP_415571) are shown aligned. Alignment was performed with ClustalW, totally conserved amino acids are highlighted with a red background, conserved hydrophobic positions with a black background. *B*, Unrooted Maximum Likelihood tree of the sequences aligned in (*A*). Sequences were aligned with MUSCLE. *C*, Structure of ciprofloxacin.

A

```
                           *        20         *        40         *        60
FnovFigB : -------MNNHIKAQILLLCFCQLIIIAAMEMSNPFLPIYLQSLGDFDLLPVNAWNVLAYAM :  55
LpneLbtB : -------MNKLVQKMMLSG---QFLMILALEMTNPFLPLLIAHQSNTPVPSVVIYNTLSLVL :  52
RpicMFS  : --MSPATPVDAWRGAIRWLLVIQALSMGAMEMTGPFWPLFLRELLPVPHVPFAWVASAAYFA :  60
EcolMdtG : MSPCENDTPINWKRNLIVAWLGCFLTGAAFSLVMPFLPLYVEQLGVTGHSALNMWSGIVFSI :  62

                         *        80         *       100         *       120
FnovFigB : PLISAMLFSPFWGKYADRFGYKTMILRAALALAIIQLLIYLTNSALLFVILRFIQGAIAGFL : 117
LpneLbtB : PMLANILLTPIWGLAADRYGYKPMLMRASWALVLTQASMIFVNSVFWILIIRLLQGAFAGFL : 114
RpicMFS  : PMAATLVTAPWWGRLADRVGPKPMILRALIALAASQLWSVYADSAASVLLARTVQGGLAGFL : 122
EcolMdtG : TFLFSAIASPFWGGLADRKGRKLMLLRSALGMGIVMVLMGLAQNIWQFLILRALLGLLGGFV : 124

                       *       140         *       160         *       180
FnovFigB : LAAQSYAVVIVSKEFRSRILAWLQSATAIGIAIGPLVGGILATLLSYKQIFLICAIIACCIF : 179
LpneLbtB : VAMQTYALSITEWQSKSTQLSRLQSSKAIATSTAGFLGGLALSFTNYQGLFGLATLICLGTT : 176
RpicMFS  : AAAQIYAMRVAPADMRRRVFADLQTVTACGSFMAPPVGAWLVGWMGFRMANTAGAAVILACI : 184
EcolMdtG : PNANALIATQVPRNKSGWALGTLSTGGVSGALLGPMAGGLLADSYGLRPVFFITASVLILCF : 186

                     *       200         *       220         *       240
FnovFigB : IILIIKLESISNPSLLSKQDNNKNCYHKNTSNNYLIYVYFSVIFLAILLSQTAKFLPQSFFA : 241
LpneLbtB : VVMHYKLPSPPKQQIKKRSQTLNHSYTSKS--------VFFFLCVLIMLTQIAKFLPDPGFT : 230
RpicMFS  : PLAMFCLPAIKPAPKSEIHQDADARRAGRAPLG----GLVIGLLLGMVAVQAARVMPQGFLA : 242
EcolMdtG : FVTLFCIREKFQPVSKKEMLHMREVVTSLKNPK-----LVLSLFVTTLIIQVATGSIAPILT : 243

                   *       260         *       280         *       300         *
FnovFigB : IYAKEFFS---SDPIIIAAIYAAPAFSLLLFSAPIGHLFDKLLNKQVNNQYFFASSYFIILF : 300
LpneLbtB : LYLNKYCS---NNLVLIGFLYSLPAMGMLCSSVWCGKQFDYCRSQPS-----LVNQYLIRYS : 284
RpicMFS  : PYITETLH---GSVMLAGLAYSATAATLALSARWWAKRFASLPVHVT-------LGRVCALT : 294
EcolMdtG : LYVRELAGNVSNVAFISGMIASVPGVAALLSAPRLGKLGDRIGPEKI----------LITAL : 295

                     320         *       340         *       360         *
FnovFigB : AISTVAIYVHGFTHNIYLILAARFILGVTFAGTLPCLFSLACRTKDNN-FGFLIGYCNTFSK : 361
LpneLbtB : VFGAILMIIQANVDNFYLFALIRILWGIVLAALLPALFALCS-DRNLL-PGYALGLANSFAK : 344
RpicMFS  : AVCGLTALWQGIAQGENAFIAARLAWGLCLGGLLPVLNSLVVETSPEHRQGFALGLCSSAAK : 356
EcolMdtG : IFSVLLLIPMSYVQTPLQLGILRFLLGAADGALLPAVQTLLVYNSSNQIAGRIFSYNQSFRD : 357

                   380         *       400         *       420         *
FnovFigB : FGNLCGIFLGGFIFQISSMQTVFYVTAIIYMFLVVIYICLLYLFDLKSSNFKNKELINV- : 420
LpneLbtB : LGNLIGLLLGGLFAFYLPYPAIFMIIAGIYSLFAIFVYGYDRLNVRQMTDFSNSYFNVKS : 404
RpicMFS  : GGALIGLGVAALSTGLFGWRAGFVAMAAMYLAALAALIAVRRAASPHPASAAAGTQRA-- : 414
EcolMdtG : IGNVTGPLMGAAISANYGFRAVFLVTAGVVLFNAVYSWNSLRRRRIPQVSN--------- : 408
```

B

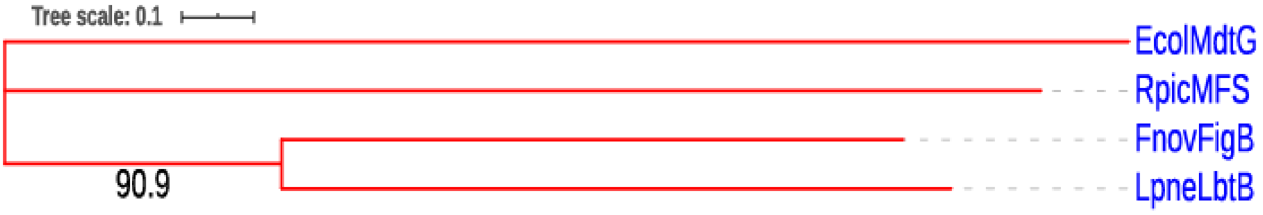

C

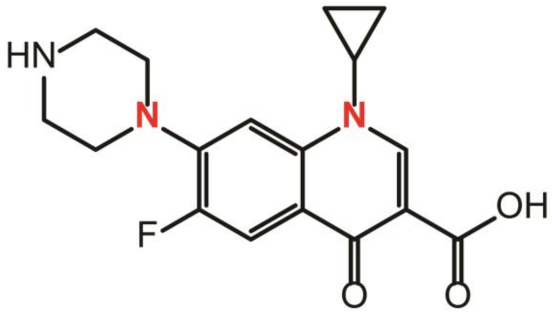

Figure S1

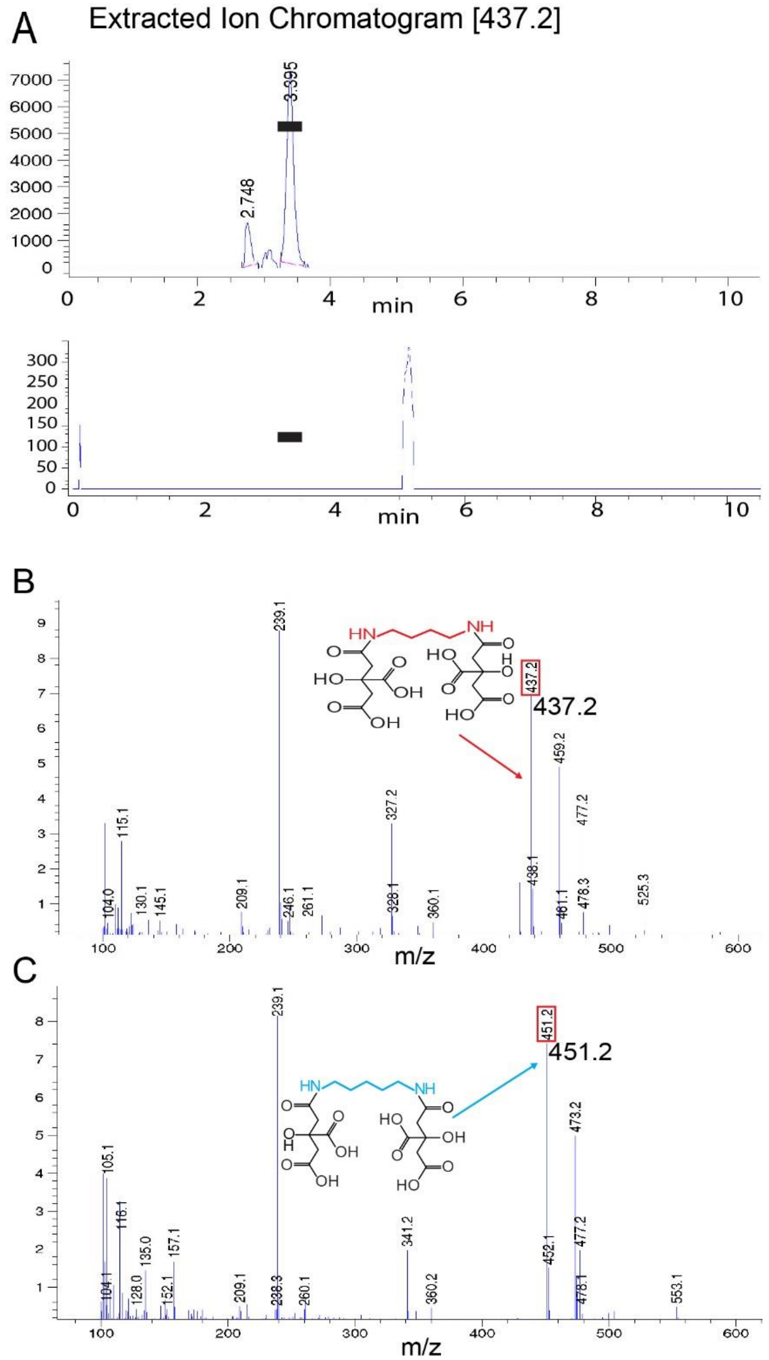

Figure S2. *In vitro* products of purified recombinant *R. pickettii* FigA homologue NIS synthetase activity detected by LC-MS. *A*, Extracted Ion Chromatograms (EIC) for the mass of rhizoferrin (437/438). Top panel, products of *R. pickettii* NIS synthetase with putrescine and Na-citrate substrates; bottom panel, no enzyme control. *B*, Mass spectrum of LC peak (3.3 min) obtained after *in vitro* incubation of *R. pickettii* NIS synthetase with putrescine. A mass corresponding to rhizoferrin is present at m/z 437.2 and its sodium adduct at m/z 459.2. *C*, Mass spectrum of LC peak obtained after *in vitro* incubation of *R. pickettii* NIS synthetase with cadaverine. A mass corresponding to homorhizoferrin is present at m/z 451.2 and its sodium adduct at m/z 473.2.

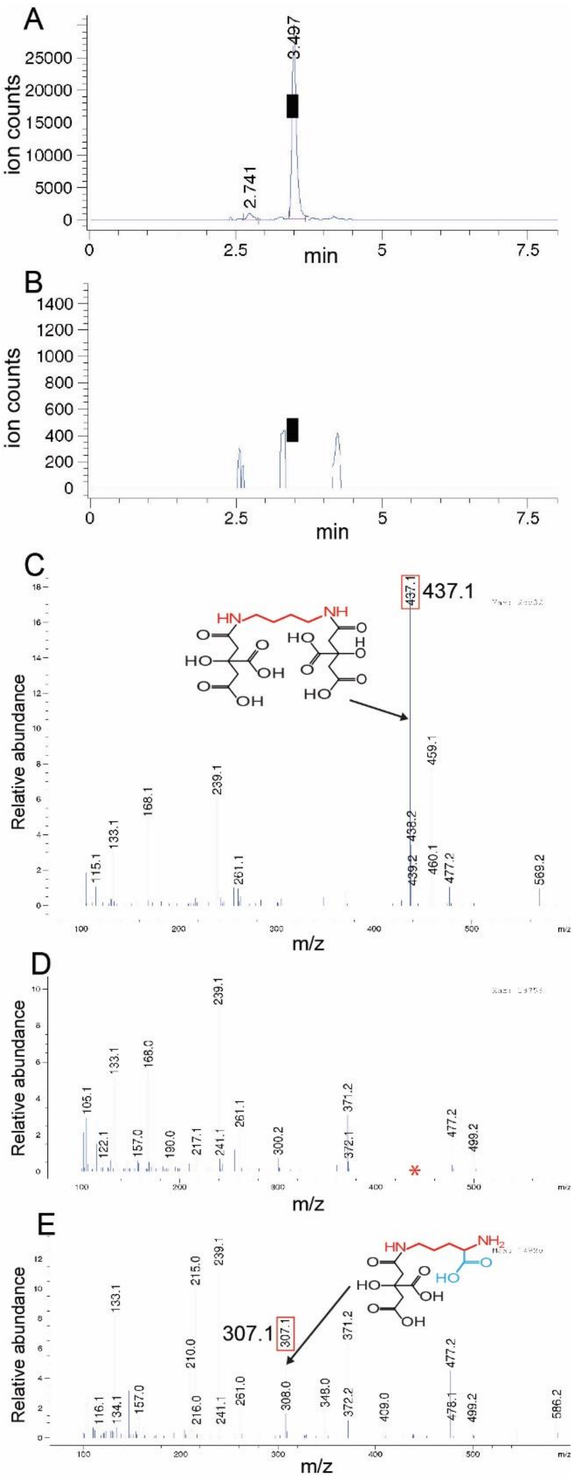

Figure S3. Mass spectra of *in vitro* products of purified recombinant *F. novicida* FigA and FigC activity. *A*,*B*, Extracted Ion Chromatograms (EIC) for the mass of rhizoferrin (437/438). *A*, Products of *R. pickettii* NIS synthetase with putrescine and Na-citrate substrates; *B*, no enzyme control. *C*, Products of FigA and FigC activity with L-ornithine and Na-citrate. A mass for rhizoferrin (437.1) and its sodium adduct at m/z 459.1 are present. *D*, No enzyme control with L-ornithine and Na-citrate. The red asterisk indicates the absence of a mass for rhizoferrin. *E*, Products of FigA in the absence of FigC with L-ornithine and Na-citrate. A mass for rhizoferrin is absent and a mass for citrylornithine (m/z 307.1) is present.
